# Examining the validity and utility of two secondary sources of food environment data against street audits in England

**DOI:** 10.1186/s12937-017-0302-1

**Published:** 2017-12-20

**Authors:** Emma L. Wilkins, Duncan Radley, Michelle A. Morris, Claire Griffiths

**Affiliations:** 1 Carnegie, Leeds Beckett University, Headingley Campus, Headingley, Leeds, LS6 3QS UK; 2 Leeds Institute for Data Analytics, School of Medicine, University of Leeds, Leeds, LS2 9JT UK

**Keywords:** ‘Retail food environment’, Validity, ‘Street audit’, Foodscape, ‘Secondary data’, ‘Obesogenic environments’, Sensitivity, ‘Positive predictive value’, ‘Administrative data’, ‘Commercial business list’

## Abstract

**Background:**

Secondary data containing the locations of food outlets is increasingly used in nutrition and obesity research and policy. However, evidence evaluating these data is limited. This study validates two sources of secondary food environment data: Ordnance Survey Points of Interest data (POI) and food hygiene data from the Food Standards Agency (FSA), against street audits in England and appraises the utility of these data.

**Methods:**

Audits were conducted across 52 Lower Super Output Areas in England. All streets within each Lower Super Output Area were covered to identify the name and street address of all food outlets therein. Audit-identified outlets were matched to outlets in the POI and FSA data to identify true positives (TP: outlets in both the audits and the POI/FSA data), false positives (FP: outlets in the POI/FSA data only) and false negatives (FN: outlets in the audits only). Agreement was assessed using positive predictive values (PPV: TP/(TP + FP)) and sensitivities (TP/(TP + FN)). Variations in sensitivities and PPVs across environment and outlet types were assessed using multi-level logistic regression. Proprietary classifications within the POI data were additionally used to classify outlets, and agreement between audit-derived and POI-derived classifications was assessed.

**Results:**

Street audits identified 1172 outlets, compared to 1100 and 1082 for POI and FSA respectively. PPVs were statistically significantly higher for FSA (0.91, CI: 0.89–0.93) than for POI (0.86, CI: 0.84–0.88). However, sensitivity values were not different between the two datasets. Sensitivity and PPVs varied across outlet types for both datasets. Without accounting for this, POI had statistically significantly better PPVs in rural and affluent areas. After accounting for variability across outlet types, FSA had statistically significantly better sensitivity in rural areas and worse sensitivity in rural middle affluence areas (relative to deprived). Audit-derived and POI-derived classifications exhibited substantial agreement (*p* < 0.001; Kappa = 0.66, CI: 0.63–0.70).

**Conclusions:**

POI and FSA data have good agreement with street audits; although both datasets had geographic biases which may need to be accounted for in analyses. Use of POI proprietary classifications is an accurate method for classifying outlets, providing time savings compared to manual classification of outlets.

**Electronic supplementary material:**

The online version of this article (doi: 10.1186/s12937-017-0302-1) contains supplementary material, which is available to authorized users.

## Background

Policymakers are increasingly recognising the role of the environment in driving obesity and associated health outcomes [1–3]. The ‘retail food environment’, characterised by the number, location and accessibility of food outlets within local environments, has been repeatedly targeted as a lever to tackle obesity [4–7]. However, evidence supporting these interventions is mixed, and predominantly null [8].

Research investigating the links between the retail food environment and obesity-related outcomes commonly uses data on food outlet locations to measure food access [9]. Access is measured using numerous spatial metrics such as density or proximity, with the majority of research investigating access to certain *types* of food outlet (e.g. ‘fast food outlets’ or ‘supermarkets’) hypothesised to have either a positive or negative effect on diet or weight status. Data on food outlet locations can be obtained through street audits; however, for efficiency reasons, it is more commonly obtained from secondary sources. The validity of these secondary data is an important consideration, repeatedly noted by authors as a limitation of these study designs [10–12]. Poor quality data can lead to uncertainty, bias, and reduced statistical power; potentially helping explain the mixed and predominantly null findings in retail food environment-obesity research. Indeed, a recent study found that the use of different data sources (from InfoUSA, and Dunn and Bradstreet) led to differences in both the strength and number of statistically significant associations between food outlet density and area-level demographics [13].

Recently, there has been increasing interest in the validity of secondary food environment data, which is typically assessed against the ‘gold standard’ of street audits [14, 15]. The vast majority of research originates from the US, wherein validity has been found to vary between different data sources, and across outlet types and environmental characteristics (e.g. deprivation and urbanicity) [14]. Overall, the percentage of food outlets captured in various US data sources has been found to range from 38% to 98% [16]. However, relatively little evidence exists in relation to the validity of UK-specific data.

Two very commonly used data sources in UK research are Ordnance Survey Points of Interest data (‘POI data’) [10–12, 17–19] and food hygiene data from local authorities [20–27]. Food hygiene data are collected by the Environmental Health department of each authority and comprise locational and business type information for all businesses engaged in ‘food operations’ (i.e. selling, cooking, storing, handling, preparing or distributing food/drink). Food hygiene data are often presented as a valid representation of the UK foodscape [24, 26, 28]; although these data have only been validated in three studies [29–31], which had relatively small sample sizes (ranging from 19 to 617) and limited geographic scope, restricting generalisability to the UK as a whole. In particular, two of these studies [29, 31] validated data within only one local authority (Newcastle and Glasgow respectively), and the third [30] validated data within three local authorities (Northumberland, Sunderland and Durham), but the audits only spanned 6 small sample areas. Given that food hygiene data are collected independently by local authorities, data quality may vary across authorities. Additionally, there is evidence that the validity of food environment data from other countries may vary across urban/rural and socioeconomic contexts [14, 16]. Geographic context is therefore important in establishing the validity of food hygiene data, and further investigation is needed across a broader range of contexts.

Historically, food hygiene data had to be requested separately for each local authority [32]. However, these data are now available centrally for all UK local authorities via the Food Standards Agency (FSA) website [33]. Personal communications with environmental health officers have indicated that there may be some differences between data obtained from the FSA and data obtained directly from local authorities (e.g. in relation to the scope of the data) meaning the validity of data obtained from the FSA website (hereinafter referred to as ‘FSA data’ to distinguish from ‘local authority data’ obtained directly from local authorities) may differ from that obtained directly from local authorities. While the food outlet data on the FSA website is updated daily, it is unclear how regularly local authorities update their own records, which would impact the validity of both the FSA and local authority data. In view of the above, validation of FSA data is needed.

POI data contains locational and classification information on over 4 million points of interest (e.g. businesses and public facilities) across the UK [34]. As well as being prominent in research, it is also used in emerging policy tools, such as the Food Environment Assessment Tool (FEAT) and the Public Health England fast food map [35, 36]. However, it has only been evaluated in one study [28], which was of limited geographic scope, and did not compare the data to the ‘gold standard’ of street audits. Thus, validation of this important dataset over a broader geographic scope, and against street audits is needed. Validation of both FSA and POI data against the same street audit data will also enable comparison between these two important datasets.

The aim of this study is to validate POI and FSA data against street audits in England. A first objective is to establish the overall agreement between the audits and the POI and FSA data respectively. As the validity of US data sources has been found to vary across outlet types and environmental characteristics a second objective is to determine whether the agreement of the POI or FSA data varies across different environment types (characterised by deprivation and urbanicity) or outlet types. As POI data includes detailed proprietary outlet classifications that have been previously used to define outlet types [10], a third aim is to establish the accuracy of POI-derived outlet classifications relative to audit-derived classifications. Finally, insights into the utility of the data are presented in order to help researchers and policymakers make a fully-informed decision around which (if any) of the two data sources to use.

## Methods

### Audit area selection

Audit areas were selected from within four local authorities in England: Leeds (having a range of urban areas with a spread of deprivation levels), Durham (having a range of rural, deprived areas), North Kesteven and Calderdale (both having a range of rural areas of middle/high affluence). There are 327 local authorities in England. Lower Super Output Area (LSOA) boundaries were used to define audit areas. LSOAs are an administrative geography in the UK with a minimum population of 1000 [37]. LSOA boundary data was obtained from the UK Data Service [38].

LSOAs were selected across six environment types: ‘urban deprived’, ‘urban middle affluence’, ‘urban affluent’, ‘rural deprived’, ‘rural middle affluence’ and ‘rural affluent’. Urban/rural designations were applied using Office for National Statistics Rural Urban Classifications at the LSOA level [38] as defined in Table 1. Deprivation designations were applied based on English Index of Multiple Deprivation (IMD) rankings [39]. As the degree of deprivation in England is not evenly distributed across urban and rural areas (e.g. only 0.8% of rural LSOAs, versus 12.0% of urban LSOAs are within the lowest decile of deprivation), LSOAs were stratified by urban/rural designation, and were re-ranked for deprivation relative to all other LSOAs with the same rural/urban classification (Additional file 1). For urban and rural areas separately, the new deprivation rankings were divided into deciles, and environment designations were applied (see Table 1).

**Table 1:** Definitions of the Six Environment Types

Environment Type	IMD Deciles^a^	Rural/Urban Classifications
Urban Affluent	urban IMD deciles 8–10	A1, B1, C1, C2
Urban Middle Affluence	urban IMD deciles 4–7	A1, B1, C1, C2
Urban Deprived	urban IMD deciles 1–3	A1, B1, C1, C2
Rural Affluent	rural IMD deciles 8–10	D1, D2, E1, E2
Rural Middle Affluence	rural IMD deciles 4–7	D1, D2, E1, E2
Rural Deprived	rural IMD deciles 1–3	D1, D2, E1, E2

*Note*. A1: Urban major conurbation; B1: Urban minor conurbation; C1: Urban city and town; C2: Urban city and town in a sparse setting; D1: Rural town and fringe; D2: Rural town and fringe in a sparse setting; E1: Rural village and dispersed; E2: Rural village and dispersed in a sparse setting; *IMD* Index of multiple deprivation

^a^IMD deciles were calculated separately for urban and rural environments as described in the main text

LSOAs were selected for auditing based on the ease with which they could be reached by the audit team and the number of expected outlets within each LSOA, as indicated by the POI data; with higher numbers chosen preferentially. LSOAs were selected to ensure at least 100 food outlets were expected within each of the six environment types (e.g. ‘rural deprived’). All LSOAs were eligible for selection. Overall, 52 LSOAs were selected for auditing (Additional file 1).

### Street audits

The boundaries of the selected LSOAs were copied by hand onto printed street maps [40–42] to define audit areas. Some small modifications were made to the LSOA boundaries for practicality reasons (see Additional file 1 for details). All streets falling within each audit area were walked and the name, street name, and outlet classification of all food outlets were recorded, forming an ‘Audit List’ of food outlets. Food outlets within private premises (e.g. members’ clubs or workplaces) or outlets not visible from the roadside (e.g. cafes within hospitals or sports centres) were not recorded.

Outlets were designated one of seven outlet types (‘Restaurant’, ‘Pub’, ‘Cafe’, ‘Fast Food’, ‘Supermarket’, ‘Convenience’, and ‘Speciality’) as defined based on the classification scheme of Lake et al. [29] (Additional file 1). All audits were performed by one of two teams of trained auditors and took place in September and October 2016. To assess inter-rater agreement, four LSOAs were audited independently by both sets of auditors.

### Secondary data

**Fig. 1:**
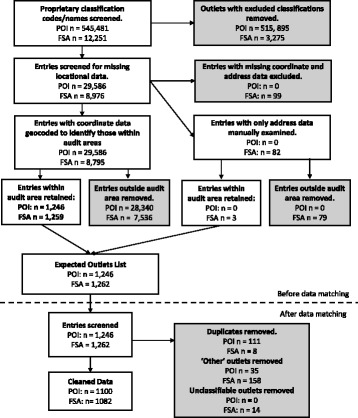
Flow chart detailing data processing procedure. POI: Points of Interest data; FSA: Food Standards Agency Data

The most recent version of POI data available at the time of the street audits was downloaded from Edina Digimap (Leeds: March 2016 version [43]; all other areas: June 2016 version [44]). The FSA data was downloaded from the Food Standards Agency website [33] on 8th December 2016. A flow chart detailing data processing steps in respect of these data is shown in Fig. 1.

Firstly, food outlets with the proprietary classification codes listed in Table 2 were extracted from each dataset (POI: *n* = 29,586; FSA: *n* = 8,976; full classification schemes for each dataset available in Additional file 1). The two datasets were then screened for missing coordinate data and/or address data. Entries missing both coordinate and address data (FSA: *n* = 99; POI: *n* = 0) were deleted, and those missing coordinate data only (FSA: *n* = 82; POI: *n* = 0) were inspected to establish whether the address fell within an audit area (FSA: *n* = 3). The remaining food outlets were plotted in ArcMap 10.4 using their associated coordinate data to identify outlets falling within the audit areas. This generated a list of expected outlets (the ‘Expected Outlets List’) for the POI and FSA data respectively.

**Table 2:** POI and FSA classification codes used to extract food outlets from the original dataset

POI classification codes (classification name)	FSA classification names^a^
1020013 (cafés, snack bars and tea rooms) 1020025 (internet cafés) 9470699 (convenience stores and independent supermarkets) 10540737 (petrol and fuel stations) 1020043 (restaurants) 1020034 (pubs) 1010006 (hotels, motels, country houses and inns) 9470662 (butchers) 9470665 (delicatessens) 9470666 (fishmongers) 9470668(green and new age goods) 9470669 (grocers, farm shops and pick your own) 9470670 (herbs and spices) 9470672 (organic, health, gourmet and kosher foods) 7400524 (baking and confectionery) 9470663 (confectioners) 9470819 (supermarkets) 9470667 (frozen foods) 1020018 (fast food and takeaway outlets) 1020019 (fast food delivery services) 1020020 (fish and chip shops) 9470661 (bakeries) 4250312 (nightclubs) 9470705 (markets)	“Pub/Club” “Restaurant/Café/Canteen” “Retailers – Supermarkets/Hypermarkets” “Retailers – Smaller” “Retailers”^b^ “Retailers – Other” “Takeaway” “Primary Producer” “Distributors/Transporters” “Manufacturers/Packers” “Hotel /Guest House”

^a^Classification names listed are the official classifications as provided in the local authority Enforcement Monitoring System documentation [55]. These names deviate slightly from the actual classification names applied to the data used in the present study, as detailed in the Supplementary Materials

^b^The ‘Retailers – Smaller Retailers’ classification is listed for completeness. However, for the data included in the present study, no food outlets had been classified within this category, with the ‘Retailers – other’ category appearing to be applied instead

### Data matching

In order to assess agreement between the audits and the POI and FSA data, entries within the Expected Outlets List for the POI and FSA data respectively were compared to the Audit List to identify matches. Matches were coded as true positives. All un-matched outlets within the Expected Outlet Lists were coded as false positives and all un-matched outlets within the Audit List were coded as false negatives.

Two separate matching criteria were utilised; referred to herein as ‘strict’ and ‘relaxed’ criteria, both mirroring matching criteria that have been employed in previous validation studies [28, 45, 46]. Under the strict matching criteria, matches were established if outlet names and street names were the same or similar. Naming discrepancies were allowed if they were grammatical e.g. ‘The Cod Father’ and ‘The Codfather’ or when the names and classifications were substantially similar (e.g. ‘Magic Wok’ and ‘Mr Wong’s Magic Wok’, both classified as ‘Restaurant’). Discrepancies in street name were allowed if an outlet was located at a junction (and could therefore have multiple legitimate street addresses) or if the outlet was on a street having multiple names (e.g. ‘Armley Road’ merging into ‘Canal Street’, Additional file 1). The ‘strict’ criteria are relevant to study designs that utilise store names in analyses e.g. to extract food outlets. However, typically retail food environment research investigates access to certain *types* of food outlets (e.g. ‘fast food outlets’), and for much of this research, outlet *names* are inconsequential. Thus, under the ‘relaxed’ matching criteria, outlet names were allowed to differ, and a match was instead required between outlet classifications and street names. Thus, outlets that had different names e.g. ‘Eastern Delight’ and ‘Double Dragon’, but the same outlet classification (‘Fast Food’), and were located on the same street were considered a match.

After data matching, the entries were manually screened to identify and subsequently remove duplicates (additional details in Additional file 1). For the POI data, 111 entries (8.9%) were removed as duplicates. For the FSA data, 8 entries (0.6%) were removed as duplicates.

Entries coded as false positives were additionally examined to assign one of the seven outlet classifications defined above, using a combination of the outlet’s proprietary classification, outlet name, and Google searching. Outlets falling outside the seven classifications additionally fell outside the scope of the street audits (e.g. childcare centres and workplace canteens), and were classified as ‘other’ and excluded. For the POI data, 35 entries (2.8%) were determined to be ‘other’-type outlets, compared to 158 (12.5%) for the FSA data. It was possible to assign a classification to all false positive entries in the POI data. However, 14 (1.1%) of the outlets in the FSA data were unclassifiable because the businesses could not be identified online. These outlets were also excluded.

### Agreement between POI-derived and audit-derived classifications

As mentioned above, the POI data includes very detailed proprietary outlet classifications, which have been used to define outlet types in research. This process was simulated in this study, with ‘POI-derived’ classifications being defined as shown in Table 3. These classifications were applied to all true positives, to allow comparison with the audit-derived classifications. Agreement between FSA classifications and audit-derived classifications was not assessed because the proprietary classifications in the FSA data lacked sufficient detail for comparison with the audit classifications.

**Table 3:** POI-derived classification scheme

Classification Name	POI Codes
Restaurant	1,020,043 (restaurants) 1,020,034 (pubs – manual Google search to identify those serving food) 1,010,006 (hotels, motels, country houses and inns)
Pub	1,020,034 (pubs) 4,250,312 (nightclubs)
Café	1,020,013 (cafés, snack bars and tea rooms) 1,020,025 (internet cafés)
Fast Food	1,020,018 (fast food and takeaway outlets) 1,020,019 (fast food delivery services) 1,020,020 (fish and chip shops) 9,470,661 (bakeries)
Supermarket	9,470,699 (convenience stores and independent supermarkets)^a^ 9,470,819 (supermarkets) 9,470,667 (frozen foods)
Convenience	9,470,699 (convenience stores and independent supermarkets)^a^ 10,540,737 (petrol and fuel stations)
Specialty	9,470,662 (butchers) 9,470,665 (delicatessens) 9,470,666 (fishmongers) 9,470,668 (green and new age goods) 9,470,669 (grocers, farm shops and pick your own), 9,470,670 (herbs and spices) 9,470,672 (organic, health, gourmet and kosher foods), 7,400,524 (baking and confectionery) 9,470,663 (confectioners)

*Note*. *POI* Points of Interest data

^a^Outlets with this classification were coded as ‘supermarket’ if they were a small format major national chain supermarket (Tesco Express, Sainsbury’s Local, M & S Simply Food, Little Waitrose and Co-operative). Otherwise, the outlets were classified as convenience stores

### Statistical analyses

All statistical analyses were conducted in R (v 3.2.3). The threshold for statistical significance was set at *p* < 0.05. All results presented are for ‘relaxed’ matching criteria (requiring a match on outlet classifications and street addresses, but not outlet names as described above), unless expressly stated.

Inter-rater agreement was assessed by comparing counts of outlets identified in the audit areas. Percentage agreement and the Kappa statistic were used to assess agreement between broad outlet classifications.

**Fig. 2:**
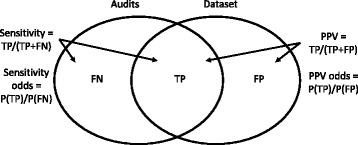
Venn diagram illustrating the classification of outlets as true positives (TP), false positives (FP) and false negatives (FN). The left-hand oval represents all outlets identified in the audits, and the right-hand oval represents all outlets identified by the secondary data (POI or FSA). The region of overlap depicts outlets that were identified in both the audits and the dataset. The figure also shows the equations used to calculate sensitivity statistics and positive predictive values (PPV) and their respective odds, where P(X) represents the probability of event X

Traditional measures of agreement for categorical data (e.g. the Kappa statistic) cannot be used to assess agreement with the street audits, because the number of ‘true negatives’ (i.e. outlets found neither in the audits nor the secondary data) is undefined. Agreement between the secondary datasets (POI and FSA) and the audits was therefore assessed via sensitivity statistics and positive predictive values (PPV); defined as shown in Fig. 2. Sensitivity statistics indicate the prevalence of missing outlets within the POI and FSA data, whereas PPV statistics indicate the prevalence of ‘erroneous’ food outlets within these data. Clopper-Pearson ‘exact’ 95% confidence intervals (CI) were calculated for sensitivities and PPVs [47].

To assess variation in agreement across environment and outlet types, PPVs and sensitivities were modelled using separate respective random intercepts multi-level logit models to account for the multi-level sampling approach used in this study (outlets nested within LSOAs). PPVs and sensitivities were treated as respective binary outcomes (sensitivity: true positive vs false negative; PPV: true positive vs false positive). Thus, the resultant odds derived from these models can be interpreted as indicating the odds of an outlet listed in the secondary dataset being a true positive versus a false positive (PPV odds) and the odds of an outlet found in the audits being a true positive versus a false negative (sensitivity odds) (Figure 2).

A series of models were run to estimate the associations between urbanicity, deprivation and outlet type, and PPVs and sensitivities. In Model 1, urbanicity was included as a single fixed effect to determine whether PPVs or sensitivities vary across urban/rural environments. In Model 2, urbanity was replaced with deprivation, to explore variation in PPVs or sensitivities across deprivation levels.

Variability in data quality across environment types may be explained by inherent geographic biases. However, it may also be explained by variation in data quality across outlet types, and differing food outlet composition across environment types (e.g. if fast food outlets have high PPVs/sensitivities then areas with higher concentrations of fast food outlets, such as deprived urban areas, will appear to have higher PPVs/sensitivities). To explore whether differing food outlet composition explains any observed geographic biases, Model 3 included urbanicity, deprivation and outlet type as fixed effects in a single model. An interaction between urbanicity and deprivation was also included to account for the dependency of deprivation on urbanicity.

Agreement between audit-derived and POI-derived classifications was compared using percentage agreement and Cohen’s Kappa statistic.

## Results

### Inter-rater agreement

Across the four LSOAs audited by both audit teams, the first identified 115 outlets and the second identified 109 (88.2% agreement). Percentage agreement for outlet classifications was 88.6%, and Kappa agreement was 0.86 (CI: 0.78–0.94), which is considered ‘almost perfect’ according to Landis and Koch [48].

### Overall agreement with audits

#### Counts of outlets

Overall, 1172 outlets were identified in the street audits, compared to 1100 and 1082 in the POI and FSA data respectively (Table 4). Both datasets under-represented the total count of food outlets across most environment and outlet types compared to the street audits. As exceptions to this, the count of outlets in middle deprived areas was equal in the audits and POI data. Additionally, pubs were over-represented in both the POI and FSA datasets (9.5% and 4.8% respectively), and supermarkets were over-represented by the POI dataset (8.6%). Counts of outlets across each local authority and LSOA are reported in Additional file 1. Counts of outlets identified in the audits ranged from 1 to 176 at the LSOA level, and from 73 to 795 at the local authority level.

**Table 4:** Counts of outlets and corresponding positive predictive values and sensitivities

Environment/Outlet Type	Audits	POI			FSA		
	Count	Count	PPV	Sens	Count	PPV	Sens
Total	1172	1100	0.86	0.81	1082	0.91	0.84
Urban	742	729	0.83	0.82	680	0.91	0.83
Deprived	249	244	0.83	0.81	225	0.91	0.82
Middle	342	344	0.81	0.81	319	0.90	0.84
Affluent	151	141	0.91	0.85	136	0.92	0.83
Rural	430	371	0.91	0.78	402	0.92	0.86
Deprived	173	161	0.86	0.80	172	0.91	0.91
Middle	135	114	0.93	0.79	122	0.91	0.82
Affluent	122	96	0.97	0.76	108	0.95	0.84
Restaurant	306	288	0.91	0.86	283	0.95	0.88
Pub	63	69	0.65	0.71	66	0.73	0.76
Café	194	152	0.87	0.68	175	0.89	0.80
Fast Food	299	299	0.87	0.87	280	0.96	0.90
Supermarket	81	88	0.82	0.89	76	0.97	0.91
Convenience	115	103	0.83	0.75	111	0.80	0.77
Specialist	114	101	0.86	0.76	91	0.92	0.74

*Note*. *Sens* sensitivity, *PPV* positive predictive value. *POI* Points of Interest. *FSA* Food Standards Agency

#### PPV and sensitivities

**Fig. 3:**
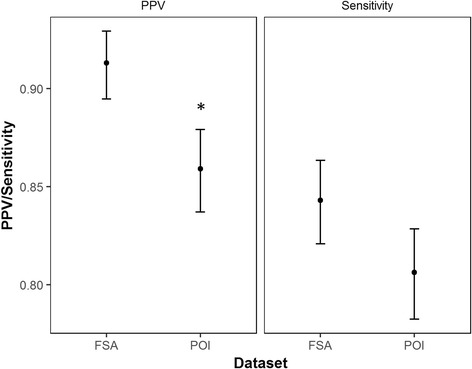
Positive Predictive Values (PPV) and sensitivities for FSA and POI data. * statistically significant difference between datasets (*p* < 0.05). FSA: Food Standards Agency data. POI: Points of Interest data. PPV: positive predictive values

Overall, the PPV was statistically significantly higher for FSA data (0.91, 95% confidence interval (CI): 0.89–0.93) than for POI data (0.86, CI: 0.84–0.88, *p* < 0.05, Figure 3). There was no statistically significant difference in sensitivity values between the two datasets (POI: 0.81, CI: 0.78–0.83; FSA: 0.84, CI: 0.82–0.86). Both the FSA and POI data had ‘good’ agreement with street audits according to the classification system of Paquet et al. [49].

When strict matching criteria were applied (i.e. requiring a match based on outlet name), PPV and sensitivity values were lower than under the relaxed matching criteria (POI: PPV: 0.79, CI: 0.77–0.82; sensitivity: 0.74, CI: 0.72–0.77; FSA: PPV: 0.87, CI: 0.85–0.89; sensitivity: 0.81, CI: 0.78–0.83).

### Variation by environment and outlet type

#### POI data

For the POI data, PPV odds varied statistically significantly across deprivation and urbanicity. In rural areas, the odds of an outlet listed in the POI data being present in reality (a ‘true outlet’) were 2.07 (1.18–4.02) times higher than in urban areas (Table 5). The odds were also 2.63 (1.34–5.43) higher in affluent areas compared to deprived areas. However, after controlling for variability in validity across outlet types, neither deprivation nor urbanicity bias remained. PPV odds varied significantly across outlet types, and were statistically significantly lower for pubs, supermarkets and convenience stores relative to restaurants.

**Table 5:** Odds of true positive relative to false positive (PPV odds) for POI data

Environment/Outlet Type	Model 1			Model 2			Model 3		
	OR	95% CI		OR	95% CI		OR	95% CI	
Urban	REF						REF		
Rural	**2.07** ^**1**^	**1.18**	**4.02**				1.31	0.69	2.61
Deprived				REF			REF		
Middle				1.08	0.58	2.05	0.78	0.39	1.41
Affluent				**2.63** ^**3**^	**1.34**	**5.43**	1.80	0.85	3.81
Restaurant							REF		
Pub							**0.19** ^**3**^	**0.09**	**0.37**
Café							0.67	0.36	1.28
Fast Food							0.66	0.37	1.16
Supermarket							**0.42** ^**1**^	**0.21**	**0.88**
Convenience							**0.39** ^**2**^	**0.19**	**0.80**
Speciality							0.56	0.27	1.21
Rural × Middle							2.69	0.89	8.40
Rural × Affluent							2.71	0.68	13.81

*Note*. *OR* Odds ratio. *CI* Confidence interval. *REF* Reference category. All models are multi-level models accounting for nesting of outlets within LSOAs

^1^ *p* < 0.05, ^2^ *p* < 0.01, ^3^ *p* < 0.001

Sensitivity odds did not vary across deprivation or urbanicity, even after controlling for variability in food outlet composition across areas (Table 6). However, sensitivity odds varied significantly across outlet types and were statistically significantly lower for pubs, cafes, convenience stores and speciality outlets relative to restaurants.

**Table 6:** Odds of true positive relative to false negative (sensitivity odds) for POI data

Environment/Outlet Type	Model 1			Model 2			Model 3		
	OR	95% CI		OR	95% CI		OR	95% CI	
Urban	REF						REF		
Rural	0.80	0.59	1.08				0.97	0.58	1.61
Deprived				REF			REF		
Middle				1.00	0.71	1.39	1.07	0.67	1.70
Affluent				1.02	0.70	1.51	1.31	0.75	2.34
Restaurant							REF		
Pub							**0.42** ^**2**^	**0.22**	**0.81**
Café							**0.36** ^**3**^	**0.23**	**0.56**
Fast Food							1.16	0.72	1.89
Supermarket							1.37	0.65	3.15
Convenience							**0.52** ^**1**^	**0.30**	**0.90**
Speciality							**0.55** ^**1**^	**0.32**	**0.98**
Rural x Middle							0.91	0.43	1.89
Rural x Affluent							0.60	0.27	1.33

*Note*. *OR*: Odds ratio. *CI*: Confidence interval. *REF* Reference category. All models are multi-level models accounting for nesting of outlets within LSOAs

^1^ *p* < 0.05, ^2^ *p* < 0.01, ^3^ *p* < 0.001

Findings were similar for the strict matching criteria, except that, for PPV odds, after adjusting for variations in agreement across outlet types, there remained a very small, but statistically significant urban/rural bias, with the odds of an outlet listed within the POI dataset being a ‘true outlet’ 1.69 (1.00 2.92) times higher in rural than in urban areas (Additional file 1). The PPV and sensitivity odds were also less variable, with supermarkets no longer statistically significantly different from restaurants for PPV odds and pubs and speciality stores no longer statistically significantly different for sensitivity odds.

#### FSA data

For the FSA data, there was no variability in PPV odds across urbanicity or deprivation, even after controlling for variability in food outlet composition across environment types (Table 7). There were, however, statistically significant variations in PPV odds across outlet types, with the odds of an outlet listed in the POI data being a ‘true outlet’ markedly lower for pubs, cafes, and convenience stores relative to restaurants.

**Table 7:** Odds of true positive relative to false positive (PPV odds) for FSA data

Environment/ Outlet Type	Model 1			Model 2			Model 3		
	OR	95% CI		OR	95% CI		OR	95% CI	
Urban	REF						REF		
Rural	1.22	0.79	1.94				1.40	0.64	3.19
Deprived				REF			REF		
Middle				0.95	0.59	1.51	0.99	0.45	2.10
Affluent				1.42	0.78	2.69	1.23	0.53	2.94
Restaurant							REF		
Pub							**0.13** ^**3**^	**0.06**	**0.28**
Café							**0.43** ^**1**^	**0.20**	**0.88**
Fast Food							1.15	0.51	2.67
Supermarket							1.97	0.52	12.93
Convenience							**0.20** ^**3**^	**0.09**	**0.42**
Speciality							0.62	0.24	1.76
Rural × Middle							0.90	0.27	3.00
Rural × Affluent							1.45	0.37	6.16

*Note*. *OR* Odds ratio. *CI* Confidence interval. *REF* Reference category. All models are multi-level models accounting for nesting of outlets within LSOAs

^1^ *p* < 0.05, ^2^ *p* < 0.01, ^3^ *p* < 0.001

In relation to sensitivity odds, Models 1 and 2 found no association with deprivation or urbanicity (Table 8). However, controlling for variability in sensitivity values across outlet types revealed a statistically significant urban/rural bias. Moreover, there was a significant interaction between deprivation and urbanicity, which after stratification of the data based on urbanicity revealed a statistically significant deprivation bias in rural areas. More particularly, the odds of a ‘true outlet’ being listed in the FSA data were 2.23 (CI: 1.21–4.28) times higher in rural than in urban areas, and among rural areas, the odds were 0.49 (CI: 0.24–0.97) times lower in middle affluence than in deprived areas. There was statically significant variation in sensitivity odds across outlet types, with ‘true’ pubs, cafes, convenience stores and speciality stores having lower odds of being listed in the FSA data than restaurants. However, after stratification of the data based on urbanity, this outlet-type variability was only evident in urban areas. All findings for the FSA data were substantively the same for the strict matching criteria (Additional file 1).

**Table 8:** Odds of true positive relative to false negative (sensitivity odds) for FSA data

Environment/Outlet Type	Model 1			Model 2			Model 3			Model 3 (urban only)			Model 3 (rural only)		
	OR	95% CI		OR	95% CI		OR	95% CI		OR	95% CI		OR	95% CI	
Urban	REF						REF								
Rural	1.27	0.86	1.88				**2.23** ^**1**^	**1.21**	**4.28**						
Deprived				REF			REF			REF			REF		
Middle				0.87	0.53	1.38	1.09	0.66	1.87	1.03	0.53	1.96	**0.49** ^**1**^	**0.24**	**0.97**
Affluent				0.85	0.52	1.36	1.01	0.57	1.77	0.94	0.49	1.77	0.60	0.29	1.26
Restaurant							REF			REF			REF		
Pub							**0.41** ^**2**^	**0.21**	**0.82**	**0.24** ^**3**^	**0.10**	**0.56**	1.26	0.37	5.85
Café							**0.56** ^**1**^	**0.34**	**0.92**	**0.47** ^**1**^	**0.25**	**0.89**	0.76	0.33	1.75
Fast Food							1.17	0.69	1.98	0.92	0.47	1.79	1.75	0.73	4.40
Supermarket							1.45	0.64	3.73	0.97	0.38	2.85	4.13	0.77	76.60
Convenience							**0.46** ^**2**^	**0.26**	**0.83**	**0.36** ^**2**^	**0.17**	**0.77**	0.68	0.27	1.75
Speciality							**0.38** ^**3**^	**0.21**	**0.67**	**0.27** ^**3**^	**0.13**	**0.56**	0.68	0.27	1.76
Rural x Middle							**0.43** ^**1**^	**0.17**	**0.98**						
Rural x Affluent							0.53	0.21	1.30						

*Note*. *OR* Odds ratio. *CI* Confidence interval. REF: Reference category. All models are multi-level models accounting for nesting of outlets within LSOAs

^1^ *p* < 0.05, ^2^ *p* < 0.01, ^3^ *p* < 0.001

### Agreement between POI-derived and audit-derived classifications

POI-derived classifications agreed with audit-derived classifications 72.2% of the time (*n* = 871) (Additional file 1), exhibiting ‘substantial’ agreement (*p* < 0.001; Kappa = 0.66, CI: 0.63–0.70) [48].

## Discussion

Secondary data on the food environment is commonly used in research and is also emergently used in policy tools [35, 36]. This study sought to validate two easily-accessible sources of UK-specific food environment data (POI and FSA) against the ‘gold-standard’ of street audits. Our key finding was that POI and FSA data both have ‘good’ agreement with street audits according to the classification system of Paquet et al. [49], providing policymakers with confidence in using research and tools based on these data.

The overall PPV was *statistically* significantly higher for the FSA data than the POI data for PPV (no difference for sensitivity). However, the magnitude of this difference is relatively small and may not substantively impact the validity of findings based on these data. Indeed, Hobbs et al. [50] compared the strength and direction of associations between food access and weight status when using POI and local authority data, and obtained similar findings for both datasets (12/12 versus 11/12 of the tested associations were null for the respective data sources).

This study used both ‘strict’ and ‘relaxed’ matching criteria, with the former requiring outlet names and street addresses to agree, and the latter being more lenient in allowing outlet names to differ, provided outlet classifications agreed. For the FSA data, agreement statistics were similar under the two matching criteria (albeit slightly lower under the strict matching). For the POI data, however, there was a more marked difference between the agreement statistics under the two matching criteria. This may indicate that the POI data are less up-to-date with changes in store *names* (but not *function*) than the FSA data. For most research, relaxed matching criteria provide the most appropriate indication of the validity of the data, because typically only the classification of an outlet is of importance, and the outlet name is not considered when deriving food access measures.

This is the first study to assess the validity of food hygiene data from the FSA. However, several studies have validated food hygiene data obtained directly from local authorities [29–31]. These found similar PPVs and sensitivities to those found in this study, with PPVs ranging from 0.79–0.92 and sensitivity values ranging from 0.60–0.95 [29–31]. This suggests that any differences in data management between the FSA and independent local authorities do not give rise to any substantive differences in data quality.

This is also the first study to assess the validity of POI data against the ‘gold standard’ of street audits. However Burgoine and Harrison [28] instead evaluated POI data against local authority data, finding a PPV of 0.75 and sensitivity of 0.60. Both values are lower than those found in the present study. It is likely that this discrepancy is due to the use of local authority data as the comparator to the POI data, rather than street audits as used in our study.

Several studies have investigated potential geographical biases in POI and local authority data [28, 30, 31]. However, these have either used small sample sizes, or have not compared the secondary data to the ‘gold standard’ of street audits, limiting the strength of their findings. Understanding geographic biases in data is important so that steps can be taken to avoid confounding; especially within the context of retail food environment research, which seeks to capture differences in the retail food environment across areas. This study found POI data to have statistically significantly higher PPV odds in rural and affluent areas (which can be interpreted as meaning that the likelihood of an outlet listed in the POI data being a ‘true outlet’ - i.e. one that exists in reality – is higher in rural than in urban areas). However, these geographic biases were entirely explained by differences in food outlet composition across these environment types. After accounting for variability in PPVs across outlet types, there was no evidence of a geographic bias. Thus, when POI data is used to study specific outlet types (e.g. fast food outlets only), geographic bias is unlikely. However, for food access metrics that consider multiple food outlet types together (e.g. fast food outlets divided by total food outlets) then geographic bias may exist.

Contrary to the present findings, Burgoine and Harrison [28] found no evidence of urban/rural bias in PPVs when comparing POI to local authority data, but did find statistically significantly lower sensitivities and percentage agreement in rural areas. However, as mentioned, Burgoine and Harrison used local authority data as reference data, and inaccuracies in the local authority data may have given rise to these different findings. Additionally, as the study area was limited to the relatively affluent and predominantly rural area of Cambridgeshire, there may have been insufficient variation in environmental characteristics to reliably detect geographic bias across the UK as a whole.

For the FSA data, there was no overall geographic bias in the PPV or sensitivity odds, which is in agreement with previous literature [30, 31]. However, after accounting for variability in agreement across outlet types, sensitivity odds were statistically significantly higher in rural than in urban areas (which can be interpreted as meaning that the likelihood of a ‘true outlet’ being listed in the FSA data is higher in rural than urban areas). Among rural areas, sensitivity odds were also lower in middle than deprived areas. This means, if FSA data is used to study specific food outlet types (as is often the case), the count of outlets may be under-estimated in urban areas relative to rural areas, and in middle affluence rural areas relative to deprived rural areas.

Many food environment studies investigate access to certain outlet types; most commonly supermarkets, convenience stores and fast food outlets [8]. Our study found that both POI and FSA data exhibited variation in both PPV and sensitivity odds across outlet types. Notably, PPV and sensitivity odds for convenience stores were low for both datasets. Low accuracy for convenience stores has also been noted in other international datasets [14], suggesting convenience store provision may be inherently difficult to capture. That said, PPVs and sensitivity values were still ‘good’ according to the classifications of Paquet et al. [49] for both datasets.

After stratifying by urbanicity, statistically significant variation in sensitivity values across the FSA data disappeared in rural environments. This is likely to be caused by smaller sample sizes within rural environments and an associated lack of power to detect significant variation across outlet types, rather than representing that sensitivity values are stable in rural environments but not in urban environments.

POI data includes approximately 24 different classification codes for food outlets, providing relatively detailed information on outlet function. The proprietary codes within the POI data have previously been used to define outlet types in research [10]. However, the accuracy with which outlets can be classified using these proprietary codes was unknown. Our study found that POI-derived classifications substantially agreed with audit-derived classifications, suggesting that use of proprietary classifications to automatically assign outlets to broad outlet classifications is a viable method for classifying outlets. This method is considerably more time-efficient than manually classifying each outlet e.g. based on Google searching, as has been carried out in other research [24, 25].

It should be noted that the reliance on outlet classifications to characterise the retail food environment is simplistic, and does not take into account food provision within individual outlets nor other factors that may influence purchasing decisions, such as pricing and preferences. However, capturing detailed features of the retail food environment such as these typically requires within-store audits, which are not practical for large-scale studies. Thus, while use of outlet classifications may not be the ‘best’ method for capturing the availability of foods within local environments, it presents a practical compromise for large-scale research.

Although FSA and POI data have been shown to be similarly valid, in our view the POI data has better utility. Firstly, POI data has more detailed proprietary outlet classifications than FSA data. It has been shown in our study that use of POI classifications to automatically assign outlets to broad outlet classifications is a viable method for classifying outlets. Conversely, for the majority of research, FSA classifications do not provide sufficient detail to characterise the retail food environment, and thus outlets must be classified via some other means e.g. use of business directories or Google searching, which is labour-intensive.

Secondly, the percentage of outlets that had to be removed from the FSA data was higher than for the POI data (14.3% vs 11.7%). Additionally, the majority of these (95.6%) were excluded as ‘other’-type (e.g. childcare centres and workplace canteens) or unclassifiable outlets, which are not usually of interest in food access studies. Conversely only a relatively small percentage (24.0%) of outlets excluded from the POI data were ‘other’-type outlets, with the remainder being duplicates. Screening for ‘other’-type outlets is thus very important for the FSA data, but less-so for the POI data. This screening process is very labour intensive, requiring all outlets to be manually classified using e.g. Google searching. Removal of duplicates from a dataset, on the other hand, is relatively simple and can be partially automated. Thus, data cleaning may be considerably more labour intensive for the FSA data.

Finally, POI data are more geographically accurate; with addresses geocoded to the address level (i.e. the precise building) [34], whereas FSA data are geocoded to the postcode level, which include multiple addresses (an average of 15 and a maximum of 100) [51]. This is illustrated in the fact that only one food outlet was missed from the POI Expected Outlets List due to a geocoding inaccuracy; whereas 16 were missed from the FSA Expected Outlets List. While it is possible to geocode the FSA data with better spatial accuracy using address look-ups, this requires additional time. Also, address information within the FSA data was sometimes missing or incomplete, meaning these addresses could not be geocoded to the address level.

Overall both datasets required considerable data cleaning. The total time taken to carry out this process was not recorded. Nevertheless, it was substantially less than the resource requirements of the street audits, which took 37 full working days and cost £555 in travel and accommodation costs, supporting the use of secondary data as an efficient means to characterise the retail food environment.

Strengths of this research included the relatively large sample sizes allowing variability in the validity of the data across outlet and environment types to be examined, and the use of ‘strict’ and ‘relaxed’ matching criteria which are applicable to different use cases that do and do not require accurate listings of outlet names. Further, in addition to data validity this study considered the utility of the data (i.e. in terms of the amount of data cleaning required, and the level of detail and accuracy of proprietary classifications); a factor that is influential in data selection.

Due to time restrictions, only four local authorities were covered in the audits. While this is an improvement over prior literature, our findings may still not be generalisable to all local authorities nationally. Additionally, as the FSA data are collected by independent local authorities, there may be variability in data quality across authorities. It is also possible (albeit less likely) that the quality of POI data varies across local authorities. To account for this, we considered including local authority as a fixed effect in our models. However, there was a high degree of correlation between local authority and urbanicity (due to the local authorities being predominantly either urban or rural, *r* = 0.84), which can lead to unstable parameter estimates [52]. We therefore chose to exclude local authority from our final model. We cannot rule out that the observed variations in data quality across urban and rural environments could also be explained by variations across local authorities.

Time and financial restrictions also meant that it was not possible to cover many ‘dispersed’ rural areas, with the majority of rural LSOAs (96.7%) being classified as ‘rural town and fringe’. Thus, results might not be generalisable to more dispersed rural environments.

Temporal mismatch between the street audits and date of acquisition of the POI and FSA data may have reduced agreement between these data and the street audits. However, the temporal mismatch was no more than 2 months, and the foodscape is unlikely to have changed substantially in this time. Additionally, temporal mismatch of this magnitude and more between exposure and outcome data is common in food access research [19, 23, 53, 54], so the present findings remain applicable to such research. It was not possible to obtain POI and FSA data from the same timeframe, and thus comparisons between the validity of the POI and FSA data may have been affected by temporal mismatch between these datasets.

Finally, the present study excluded food outlets whose primary function was not food retail from the audits e.g. department stores and entertainment venues. This was firstly because it was often not possible to establish from the roadside whether such outlets sold food, and secondly because such establishments are generally not considered in retail food environment research. However, Lucan et al. [46] found that 23.9% of outlets selling food in New York were businesses not primarily engaged with food retail. Thus, such establishments may make up an important component of the retail food environment. These establishments appear to be listed in both the FSA and POI data, although the completeness of these listings is unknown and extraction of such outlets, particularly for the POI data, will be challenging. One technique may be to extract major chain outlets not primarily engaged in food retailing but known to retail food (e.g. large pharmacies and department stores) based on outlet name. This would not capture all businesses where food retail is secondary to another service, but would present an improvement over existing techniques.

## Conclusion

The retail food environment is increasingly targeted as a lever to improve diet and reduce obesity. Food hygiene data (e.g. from local authorities or the FSA) and POI data are both frequently used in research and emergently used in policy tools to characterise the UK food environment. This study found POI and FSA data to have ‘good’ agreement with street audits. Both datasets had variable validity across outlet types and geographic biases, which may need to be accounted for in analyses. Overall policymakers can have confidence in tools and evidence based in these data, although for certain applications (e.g. when policymakers need to know locations of specific food outlets) these data may not be sufficiently valid. Presently local authorities have free access to both FSA data and POI data (via the Food Environment Assessment Tool [36]). While both datasets were similarly valid, in our view the utility of the POI data was better than the FSA data. In particular, use of proprietary classifications in POI data to define outlet classifications was shown to be an accurate method for classifying outlets, which could provide substantial time savings compared to manual classification of outlets. Both datasets required substantial data cleaning, requiring several phases (e.g. removal of duplicates, identification of ‘other’-type outlets). These are important methodological steps that impact the validity of data, and should be reported in research papers.

## Additional file

Additional file 1: Additional information on methodology and results. (DOCX 657 kb)

## Abbreviations

CI: 95% confidence interval
FSA: Food standards agency
IMD: Index of multiple deprivation
LSOA: Lower super output area
POI: Points of Interest
PPV: Positive predictive value
