# Supplementary material for: Examining the validity and utility of two secondary sources of food environment data against street audits in England
Source: Nutr J. 2017 Dec 20;16:82. doi: 10.1186/s12937-017-0302-1 (PMC5738834; doi:10.1186/s12937-017-0302-1)
Supplement: Additional file 1: — Additional information on methodology and results. (DOCX 657 kb) [file 12937_2017_302_MOESM1_ESM.docx]

**Supplementary Materials**

# Additional information on POI and FSA data

## Description of FSA Data

In the UK, any business intending to conduct ‘food operations’ (i.e. selling, cooking, storing, handling, preparing or distributing food/drink) must register the business with their local authority. This data is used for enforcing Food Safety laws and in particular for carrying out inspections of food businesses to assign food hygiene ratings. From 2009 local authorities have been required, under the Food Hygiene Information/Rating Schemes to supply food hygiene ratings data to the Food Standards Agency (FSA) for publication on their website (Food Standards Agency, 2015). The schemes require ratings data to be provided for all registered food businesses that supply food/drink directly to consumers. Notably, however, certain FO (e.g. those deemed to be low-risk; such as pharmacies) may not receive a hygiene rating and may be excluded from the FSA data. Thus, while the FSA data should comprise most FO, it may exclude certain ‘low-risk’ FO that are nevertheless important for RFE assessment.

For each food business, the FSA data includes (inter alia), business name, business address, business classification, and locational coordinates (latitude and longitude according to the WGS84 geographic Coordinate Reference System). There is no publically available information on how the locational coordinates are derived. However, the majority of locational coordinates appear to align with points generated through postcode geocoding, suggesting that the majority of coordinates are geocoded to the postcode level. The business classifications are applied by local authorities according to the ‘Local Authority Enforcement Monitoring System’ (LAEMS) classification scheme, which comprises 15 classifications (Table 1) (Food Standards Agency, 2016b).

It should be noted that there is some inconsistency in nomenclature between the official FSA classification names (listed in Table 1) and the classification names that were found to have been applied to the data as detailed:

- Primary Producer also named Farmers/Growers
- Caring Establishment also named Hospitals/Childcare/Caring Premises
- Pub/Club also named Pub/Bar/Club
- Hotel/Guesthouse also named Hotel/Bed & Breakfast/Guesthouse
- School/College also named School/College/University
- Take-away also named Takeaway/Sandwich Shop
- Mobile Food Unit also named Mobile Caterer

Local authorities are required to supply their ratings data every 27 days, and this data is then made available to the public via the FSA website. How frequently each local authority updates its own database of food businesses is unclear, and the currency of the FSA data may vary between local authorities. All 392 local authorities across the UK are presently participating in the Information/Rating schemes; however, this is not obligatory for local authorities in England and Scotland.

Table 1

*FSA Classification Names and Definitions*

| **Classification Name** | **FSA Definition/Examples** |
| --- | --- |
| Primary Producer | “Examples:   - Fruit and vegetable growers - Pick your own farms - Egg producers - Potato growers - Fish farms - Beekeepers - Vineyards” |
| **Manufacturers/**  **Packers*** | “Examples:   - Abattoirs - Brewery - Meat manufacturers - Milk processors & dairy processors - Cheesemakers - Soft drinks, mineral waters - Vegetable drying, freezing, canning - Meat or poultry cutting establishments - Purification centres for shellfish - Fish processors - Butchers shops cooking hams - Fruit & vegetable co-operatives - Egg packers - Contract packers - Food contact material and article manufacturers & suppliers - Bakers with no on-site retail - Bakeries selling through their own shops - Home cake makers selling to other businesses” |
| Importers/Exporters | “Examples:   - Warehouses for import/export purposes - Freight depots, transit sheds, stores” |
| Distributors/Transporters | “Examples:   - Food brokers - Wholesalers - Cash & carries - Cold stores - Haulage companies - Milk distributors” |
| Restaurant & Caterers -Caring Establishment | “Establishments with catering services for clients/customers who are provided with care, medical treatment, supervision, or assistance.  Examples:   - Hospitals (include each establishment but not each kitchen) - Nursing/care homes - Childcare facilities/nurseries/childminders” |
| Restaurant & Caterers -  School/College | “Catering services located within a site providing educational instruction and formal qualifications.  Examples:   - Colleges - Schools (include each establishment but not each kitchen)” |
| **Restaurant & Caterers -**  **Hotel /Guesthouse** | “Establishments that provide catering only to customers to whom they are also providing accommodation. (Hotels that provide a restaurant service to a wider clientele than their guests should be recorded under the 'restaurant/café/canteen' category).  Examples:   - Hotels - Guest houses - Bed and breakfast” |
| Restaurant & Caterers -  Mobile Food Unit | “A food establishment that comprises a kitchen or catering facility operating from a mobile unit such as a vehicle, trailer, stall, marquee or other non-permanent structure.  Examples:   - Mobile catering units - Burger vans and other fast food vans/trailers/stalls” |
| **Restaurant & Caterers -**  **Pub/Club** | “Commercial establishments that primarily serve alcohol in a public bar. If the establishment has a separate restaurant facility it should be recorded under the pub category.  Examples:   - Public Houses - Night clubs/clubs with bars” |
| **Restaurant & Caterers -**  **Restaurant/Café/Canteen** | \| “Establishments whose primary business is to cook/prepare food for consumption by customers at a seated area on the premises.  Examples:   - Restaurants - Cafés - Self-service cater - ‘Fast food' establishments providing seating, e.g. McDonalds, Burger King etc. The drive-thru variants of these chains should also be included in this category.” \| \| --- \| |
| **Restaurant & Caterers -**  **Take-away** | “Establishments that provide convenience food to customers, primarily for consumption off the premises. Establishments must be immobile and housed in a designated building  Examples:   - Fish & chip shops - Take-away - Sandwich shops - Establishments that prepare and deliver convenience food directly to the customer” |
| Restaurant & Caterers -  Other Catering Premises | “Restaurant/catering establishments that do not fit into one of the other 'restaurants and caterers' categories.  Examples:   - Home caterers such as cake makers selling directly to consumers - Village halls, community centres etc. used by charitable/community organisations, see www.food.gov.uk/enforcement/enforcework/food-law/guidance-enforcement/community-hall-guidance - Ships' catering spaces” |
| **Retailers – Supermarkets**  **/Hypermarkets** | “Supermarkets e.g. Sainsbury, Tesco, Asda, Morrison, Co-op, Marks and Spencer, Waitrose, Aldi, Lidl, Budgens etc. that provide a range of food items from more than one grocery sector and from a range of brands. Also city centre or local variants of larger supermarket groups, e.g. Sainsbury's local, Tesco Metro, Tesco Express etc.  Examples:   - Supermarkets - the large retail chains - City centre or local variants of larger supermarket groups” |
| **Retailers – Smaller Retailers**** | “Smaller-scale food businesses such as butchers, bakers, fishmongers, village shops, grocers etc. Independent retailers e.g. Costcutter, One-Stop, Londis, Nisa, Premier etc.  Examples:   - Grocers - Confectioners - Butchers (retail only) - Fishmongers - Greengrocer/fruiterer - Health food shops - Bakers shops (retail only) - Newsagents - Mobile vans (retailers) - Market stalls (retailers) - Farm shops (if farm not included under producers or other establishments) - Off licences - Garage minimarkets” |
| **Retailers - other** | “Retail establishments which do not fit into one of the other retailer categories, e.g. establishments that primarily sell non-food products and a very limited range of food products.  Examples:   - Shops where the main business is not food, e.g. chemist/pharmacy that sell cough sweets/limited range of other confectionery” |

*Note*. Classification names and definitions are taken directly from Food Standards Agency (2016b). * This classification was applied by some local authorities to retail butchers which should have been classified under ‘retailers – smaller retailers’

**This classification had not been applied to any of the data used in the present study; all outlets that should fall within this classification were instead classified as ‘Retailers – other’. Items in bold are those extracted.

## Description of POI Data

POI is a dataset detailing over 4 million geographic features across Great Britain (Ordnance Survey, 2015b). It is produced by PointX Ltd on behalf of Ordnance Survey (the national mapping agency for Great Britain) for a variety of uses, including the provision of facilities and infrastructure, driver routing and navigation, emergency planning, location-based services and tourism. While access to the data is usually at a cost, it is available for free for research purposes under an educational license, and is often used in research examining the built and natural environment (Cetateanu & Jones, 2014; Fraser, et al., 2012a; Harrison, et al., 2011; Jennings, et al., 2011; Skidmore, et al., 2010)

According to the user guide, the POI data is obtained from around 140 data suppliers, which are described as “the most authoritative source or sources for the particular type of feature they supply and for the quality and completeness of the data they supply” (Ordnance Survey, 2015b). The data suppliers provide updates at different frequencies, ranging from bi-monthly to yearly. Thus the currency of the data can vary between features.

The POI dataset contains coordinate (eastings and northings according to the British National Grid projected coordinate reference system), classification and address information for each feature therein. Feature classifications are shown at Table 15. The classification scheme comprises over 600 classifications descriptive of a feature’s function. The classifications fall within one of nine groups: "accommodation, eating and drinking”, “commercial services”, “attractions”, “sport and entertainment”, “education and health”, “public infrastructure”, “manufacturing and production”, “retail”, and “transport” (Ordnance Survey, 2013). Classifications are generally applied to the data by the original data supplier. However, PointX also apply classifications if none is provided by the data supplier. Documentation is provided by PointX detailing common names/brands of businesses falling within each classification to facilitate interpretation of the classifications.

According to the user guide, the coordinate data for each feature is derived by geocoding the feature to an address location (i.e. within a building footprint) wherever possible (79.87% of features were geocoded using this method in the September 2014 release). However when this is not possible, features are either geocoded to an adjacent address, a street segment midpoint or a geographic locality (e.g. village or industrial estate). The latter two methods are only used for a small range of feature types and are not used for food outlets. According to the user guide, 95% of features are geocoded to within 17.51 metres of their true location.

Table 2

*List of Points of Interest Classifications and Associated Groupings*

| **Group/Sub-Group** | | **Classification Name** |
| --- | --- | --- |
| Accommodation, Eating and Drinking | |  |
|  | Accommodation | Camping, caravanning, mobile homes, holiday parks and centres |
|  |  | Bed and breakfast and backpacker accommodation |
|  |  | Hostels and refuges for the homeless |
|  |  | Hotels, motels, country houses and inns |
|  |  | Self catering |
|  |  | Timeshare |
|  |  | Youth accommodation |
|  | Eating and Drinking | Banqueting and function rooms |
|  |  | Cafés, snack bars and tea rooms |
|  |  | Fast food and takeaway outlets |
|  |  | Fast food delivery services |
|  |  | Fish and chip shops |
|  |  | Internet cafés |
|  |  | Pubs, bars and inns |
|  |  | Restaurants |
| Commercial Services | |  |
|  | Construction Services | Metalworkers including blacksmiths |
|  |  | Building contractors |
|  |  | Construction completion services |
|  |  | Construction plant |
|  |  | Cutting, drilling and welding services |
|  |  | Demolition services |
|  |  | Diving services |
|  |  | Electrical contractors |
|  |  | Gardening, landscaping and tree surgery services |
|  |  | Glaziers |
|  |  | Painting and decorating services |
|  |  | Plasterers |
|  |  | Plumbing and heating services |
|  |  | Pool and court construction |
|  |  | Restoration and preservation services |
|  |  | Road construction services |
|  |  | Roofing and chimney services |
|  |  | Fencing and drystone walling services |
|  |  | Building and component suppliers |
|  | Consultancies | Architectural and building related consultants |
|  |  | Business related consultants |
|  |  | Computer consultants |
|  |  | Construction service consultants |
|  |  | Feng shui consultants, furnishers and shop fitters |
|  |  | Food consultants |
|  |  | Image consultants |
|  |  | Interpretation and translation consultants |
|  |  | Security consultants |
|  |  | Telecommunications consultants |
|  |  | Traffic management and transport related consultants |
|  | Employment and career agencies | Careers offices and armed forces recruitment |
|  |  | Domestic staff and home help |
|  |  | Driver agencies |
|  |  | Employment agencies |
|  |  | Modelling and theatrical agencies |
|  |  | Nursing agencies |
|  | Engineering services | Aviation engineers |
|  |  | Chemical engineers |
|  |  | Civil engineers |
|  |  | Electrical and electronic engineers |
|  |  | Hydraulic engineers |
|  |  | Industrial engineers |
|  |  | Instrumentation engineers |
|  |  | Marine engineers and services |
|  |  | Mechanical engineers |
|  |  | Pneumatic engineers |
|  |  | Precision engineers |
|  |  | Structural engineers |
|  | Contract services | Agricultural contractors |
|  |  | Aircraft charters |
|  |  | Catering services |
|  |  | Contract cleaning services |
|  |  | Display and window dressers |
|  |  | Drain and sewage clearance |
|  |  | Linen hire and washroom services |
|  |  | Office services |
|  |  | Packers |
|  |  | Pest and vermin control |
|  | IT, advertising, marketing and media services | Advertising services |
|  |  | Artists, illustrators and calligraphers |
|  |  | Computer security |
|  |  | Computer systems services |
|  |  | Concert/exhibition organisers and services |
|  |  | Database services |
|  |  | Desktop publishing services |
|  |  | Electronic and internet publishers |
|  |  | Film and video services |
|  |  | General computer services |
|  |  | Internet services |
|  |  | Literary services |
|  |  | Mailing and other information services |
|  |  | Marketing services |
|  |  | Plate makers, print finishers and type setters |
|  |  | Press and journalism services |
|  |  | Printing and photocopying services |
|  |  | Recording studios and record companies |
|  |  | Telephone, telex and fax services |
|  |  | Television and radio services |
|  | Legal and financial | Accountants and auditors |
|  |  | Auctioneers, auction rooms and valuers |
|  |  | Banks and building societies |
|  |  | Currency conversion and money transfers |
|  |  | Cash machines |
|  |  | Cheque cashing |
|  |  | Company registration and trademarks |
|  |  | Copyright and patent |
|  |  | Credit reference agencies |
|  |  | Debt collecting agencies |
|  |  | Financial advice services |
|  |  | Fundraising services |
|  |  | Insurers and support activities |
|  |  | Mortgage and financial lenders |
|  |  | Pawnbrokers |
|  |  | Solicitors, advocates and notaries public |
|  |  | Stocks, shares and unit trusts |
|  |  | Commodity dealers |
|  |  | Franchise and holding company services |
|  |  | Paypoint locations |
|  |  | Pension and fund management |
|  | Personal, consumer and other services | Hotel booking agencies |
|  |  | Event ticket agents and box office |
|  |  | Astrologers, clairvoyants and palmists |
|  |  | Hair and beauty services |
|  |  | Cleaning services |
|  |  | Customer service centres |
|  |  | CV writers |
|  |  | Detective and investigation agencies |
|  |  | Funeral and associated services |
|  |  | Historical research |
|  |  | Headquarters, administration and central offices |
|  |  | Introduction and dating agencies |
|  |  | Lock, key and security services |
|  |  | Message and greeting services |
|  |  | Motoring organisations |
|  |  | Party organisers |
|  |  | Personalisation |
|  |  | Photographic services |
|  |  | Sports services |
|  |  | Tattooing and piercing services |
|  |  | Trophies and engraving services |
|  |  | Vehicle cleaning services |
|  |  | Weather services |
|  |  | Wedding services |
|  |  | Window cleaners |
|  |  | Musicians, orchestras and composers |
|  |  | Sculptors, wood workers and stone masons |
|  |  | Tailoring and clothing alteration |
|  |  | Vehicle breakdown and recovery services |
|  |  | Sewage services |
|  |  | Spas |
|  |  | Slimming clubs and services |
|  |  | Adult services |
|  |  | Printing on garments |
|  | Property and development services | Commercial property letting |
|  |  | Property sales |
|  |  | Estate and property management |
|  |  | Property letting |
|  |  | Property development services |
|  |  | Property information services |
|  | Recycling services | Recycling, reclamation and disposal |
|  |  | Rag merchants |
|  |  | Clearance and salvage dealers |
|  |  | Scrap metal dealers and breakers yards |
|  |  | Waste paper merchants |
|  | Repair and servicing | Building repairs |
|  |  | Electrical equipment repair and servicing |
|  |  | Household repairs and restoration |
|  |  | Industrial repairs and servicing |
|  |  | Service industry equipment repairs |
|  |  | Sports and leisure equipment repair |
|  |  | Tool repairs |
|  |  | Vehicle repair, testing and servicing |
|  |  | Shoe repairs |
|  | Research and design | Design services |
|  |  | Research services |
|  |  | Testing and analysis services |
|  | Transport, storage and delivery | Airlines and airline services |
|  |  | Animal transportation |
|  |  | Container and storage |
|  |  | Courier, delivery and messenger |
|  |  | Distribution and haulage |
|  |  | Ferry and cruise companies |
|  |  | Import and export services |
|  |  | Railway related services |
|  |  | Removals and shipping agents |
|  |  | Taxi services |
|  | Hire services | Boat hiring services |
|  |  | Construction and tool hire |
|  |  | Leisure equipment hirings |
|  |  | Renting and leasing of personal and household goods |
|  |  | Sound, light and vision service and equipment hire |
|  |  | Vehicle hire and rental |
|  |  | Clothing hire |
|  |  | Bouncy castles and inflatables hire |
| Attractions | |  |
|  | Botanical and zoological | Aquaria and sea life centres |
|  |  | Bird reserves, collections and sanctuaries |
|  |  | Butterfly farms |
|  |  | Farm based attractions |
|  |  | Horticultural attractions |
|  |  | Salmon ladders |
|  |  | Zoos and animal collections |
|  | Historical and cultural | Archaeological sites |
|  |  | Battlefields |
|  |  | Historic buildings including castles, forts and abbeys |
|  |  | Historic and ceremonial structures |
|  |  | Historical ships |
|  |  | Museums |
|  |  | Art galleries |
|  | Recreational | Commons |
|  |  | Country and national parks |
|  |  | Picnic areas |
|  |  | Playgrounds |
|  |  | Municipal parks and gardens |
|  | Landscape features | Designated scenic features |
|  |  | Trigonometric points |
|  | Tourism | Laseria, observatories and planetaria |
|  |  | Model villages |
|  |  | Railways (heritage, steam and miniature) |
|  |  | Theme and adventure parks |
|  |  | Siteseeing, tours, viewing and visitor centres |
|  |  | Information centres |
|  |  | Unspecified and other attractions |
|  | Bodies of water | Ponds |
|  |  | Lakes and waters |
|  |  | Lochs and lochans |
|  |  | Tarns, pools and meres |
|  |  | Reservoirs |
|  |  | Settling, balancing and silt ponds |
| Sport and Entertainment | |  |
|  | Sport and entertainment support services | Children's activity centres |
|  |  | Entertainment services |
|  |  | Firework related services |
|  |  | Funfair services |
|  |  | Mobile discos |
|  |  | Motorsport services |
|  | Gambling | Amusement parks and arcades |
|  |  | Bingo halls |
|  |  | Bookmakers |
|  |  | Casinos |
|  |  | Pools promoters |
|  | Outdoor pursuits | Angling and sports fishing |
|  |  | Combat, laser and paintball games |
|  |  | Hot air ballooning |
|  |  | Parachuting and bungee jumping |
|  |  | Paragliding and hang gliding |
|  |  | Watersports |
|  |  | Riding schools, livery stables and equestrian centres |
|  |  | Outdoor pursuit organisers and equipment |
|  | Sports complex | Athletics facilities |
|  |  | Bowling facilities |
|  |  | Climbing facilities |
|  |  | Golf ranges, courses, clubs and professionals |
|  |  | Gymnasiums, sports halls and leisure centres |
|  |  | Ice rinks |
|  |  | Motorsport venues |
|  |  | Racecourses and greyhound tracks |
|  |  | Shooting facilities |
|  |  | Ski infrastructure and aerial cableways |
|  |  | Snooker and pool halls |
|  |  | Sports grounds, stadia and pitches |
|  |  | Squash courts |
|  |  | Swimming pools |
|  |  | Tennis facilities |
|  |  | Velodromes |
|  | Venues, stage and screen | Cinemas |
|  |  | Discos |
|  |  | Nightclubs |
|  |  | Social clubs |
|  |  | Theatres and concert halls |
|  |  | Conference and exhibition centres |
|  |  | Adult venues |
| Education and Health | |  |
|  | Animal welfare | Animal clipping and grooming |
|  |  | Dog training |
|  |  | Horse training |
|  |  | Kennels and catteries |
|  |  | Pet cemeteries and crematoria |
|  |  | Veterinarians and animal hospitals |
|  |  | Veterinary pharmacies |
|  | Education support services | Education authorities |
|  |  | Education services |
|  |  | Examination boards |
|  |  | Playing for success centres |
|  |  | Secure units |
|  | Health practitioners and establishments | Alternative, natural and complementary |
|  |  | Foot related services |
|  |  | Dental technicians |
|  |  | Dieticians and nutritionists |
|  |  | Homeopaths |
|  |  | Midwifery |
|  |  | Optometrists and opticians |
|  |  | Physical therapy |
|  |  | Speech therapists |
|  |  | Surgeons and cosmetic surgeries |
|  |  | Chemists and pharmacies |
|  |  | Clinics and health centres |
|  |  | Dental and medical laboratories |
|  |  | Dental surgeries |
|  |  | Doctors surgeries |
|  |  | Hospices |
|  |  | Hospitals |
|  |  | Mental health centres and practitioners |
|  |  | Nursing and residential care homes |
|  |  | Accident and emergency hospitals |
|  |  | Parenting and childcare services |
|  |  | Walk-in centre |
|  |  | Day and care centres |
|  | Health support services | Medical equipment rental and leasing |
|  |  | Ambulance and medical transportation services |
|  |  | Blood transfusion service |
|  |  | Counselling and advice services |
|  |  | Health authorities |
|  |  | Medical waste disposal services |
|  |  | Pregnancy related services and help centres |
|  |  | X-ray services |
|  | Primary, secondary and tertiary education | First, primary and infant schools |
|  |  | Further education establishments |
|  |  | Independent and preparatory schools |
|  |  | Broad age range and secondary state schools |
|  |  | Special schools and colleges |
|  |  | Higher education establishments |
|  |  | Unspecified and other schools |
|  |  | Pupil referral units |
|  | Recreational and vocational education | Ballet and dance schools |
|  |  | Beauty and hairdressing schools |
|  |  | Diving schools |
|  |  | Drama schools |
|  |  | Driving and motorcycle schools |
|  |  | First aid training |
|  |  | Flying schools |
|  |  | Language schools |
|  |  | Martial arts instruction |
|  |  | Music teachers and schools |
|  |  | Nursery schools and pre and after school care |
|  |  | Sailing schools |
|  |  | Sports and fitness coaching |
|  |  | Training providers and centres |
| Public Infrastructure | |  |
|  | Central and local government | Armed services |
|  |  | Coastal safety |
|  |  | Consular services |
|  |  | Courts, court services and tribunals |
|  |  | Driving test centres |
|  |  | Embassies and consulates |
|  |  | Fire brigade stations |
|  |  | Central government |
|  |  | Local government |
|  |  | Revenue and customs offices |
|  |  | Job centres |
|  |  | Members of parliament and members of european parliament |
|  |  | Police stations |
|  |  | Prisons |
|  |  | Probation offices and police support services |
|  |  | Registrars offices |
|  |  | Social service activities |
|  |  | Tribunals |
|  |  | Foreign country support activities |
|  | Infrastructure and facilities | Electrical features |
|  |  | Fire safety features |
|  |  | Gas features |
|  |  | Meteorological features |
|  |  | Refuse disposal facilities |
|  |  | Waste storage, processing and disposal |
|  |  | Telecommunications companies |
|  |  | Telecommunications features |
|  |  | Utility companies and brokers |
|  |  | Allotments |
|  |  | Cemeteries and crematoria |
|  |  | Drinking fountains and water points |
|  |  | Halls and community centres |
|  |  | Letter boxes |
|  |  | Libraries |
|  |  | Places of worship |
|  |  | Public telephones |
|  |  | Public toilets |
|  |  | Recycling centres |
|  |  | Wifi hotspots |
|  | Organisations | Animal welfare organisations |
|  |  | Fan clubs and associations |
|  |  | Sports clubs and associations |
|  |  | Institutes and professional organisations |
|  |  | Political parties and related organisations |
|  |  | Religious organisations |
|  |  | Youth organisations |
|  |  | Community networks and projects |
|  |  | Charitable organisations |
|  |  | Conservation organisations |
| Manufacturing and Production | |  |
|  | Consumer products | Baby, nursery and playground equipment |
|  |  | Beds and bedding |
|  |  | Brushes |
|  |  | Candles |
|  |  | Canvas goods |
|  |  | Carpets, flooring, rugs and soft furnishings |
|  |  | Medals, trophies, ceremonial and religious goods |
|  |  | China and glassware |
|  |  | Clothing, components and accessories |
|  |  | Cookers and stoves - non electrical |
|  |  | Cosmetics, toiletries and perfumes |
|  |  | Curtains and blinds |
|  |  | Cutlery and tableware |
|  |  | Disability and mobility equipment |
|  |  | Refrigeration and freezing appliances |
|  |  | Footwear |
|  |  | Furniture |
|  |  | Garden goods |
|  |  | Giftware |
|  |  | Hobby, sports and pastime products |
|  |  | Disposable products |
|  |  | Jewellery, gems, clocks and watches |
|  |  | Lampshades and lighting |
|  |  | Leather products |
|  |  | Lingerie and hosiery |
|  |  | Luggage, bags, umbrellas and travel accessories |
|  |  | Musical instruments |
|  |  | Photographic and optical equipment |
|  |  | Saunas and sunbeds |
|  |  | Tents, marquees and camping equipment |
|  |  | Tobacco products |
|  |  | Fireplaces and mantelpieces |
|  |  | Conservatories |
|  |  | Bathroom fixtures, fittings and sanitary equipment |
|  | Extractive industries | Coal mining |
|  |  | Oil and gas extraction, refinery and product manufacture |
|  |  | Ore mining |
|  |  | Peat extraction |
|  |  | Sand, gravel and clay extraction and merchants |
|  |  | Stone quarrying and preparation |
|  |  | Unspecified quarries or mines |
|  | Farming | Animal breeders (not horses) |
|  |  | Arable farming |
|  |  | Bee keepers |
|  |  | Dairy farming |
|  |  | Fish and shellfish |
|  |  | Forestry |
|  |  | Fruit, flower and vegetable growers |
|  |  | Hoppers and silos |
|  |  | Horse breeders and dealers |
|  |  | Livestock farming |
|  |  | Mixed or unspecified farming |
|  |  | Poultry farming, equipment and supplies |
|  |  | Sheep dips and washes |
|  | Foodstuffs | Alcoholic drinks |
|  |  | Animal feeds, pet foods, hay and straw |
|  |  | Baking and confectionery |
|  |  | Dairy products |
|  |  | Fish, meat and poultry products |
|  |  | Milling, refining and food additives |
|  |  | Non alcoholic drinks |
|  |  | Catering and non specific food products |
|  | Industrial features | Business parks and industrial estates |
|  |  | Chimneys |
|  |  | Conveyors |
|  |  | Energy production |
|  |  | Lighting towers |
|  |  | Lime kilns |
|  |  | Oast houses |
|  |  | Pipelines |
|  |  | Tanks (generic) |
|  |  | Travelling cranes and gantries |
|  |  | Unspecified works or factories |
|  |  | Water pumping stations |
|  | Industrial products | Abrasive products and grinding equipment |
|  |  | Adhesives and sealants |
|  |  | Aeroplanes |
|  |  | Agricultural machinery and goods |
|  |  | Air and water filtration |
|  |  | Arms and ammunition |
|  |  | Bearing, gear and drive elements |
|  |  | Beekeeping supplies |
|  |  | Bricks, tiles, clay and ceramic products |
|  |  | Cable, wire and fibre optics |
|  |  | Colours, chemicals and water softeners and supplies |
|  |  | Cleaning equipment and supplies |
|  |  | Concrete products |
|  |  | Cooling and refrigeration |
|  |  | Electrical components |
|  |  | Electrical motors and generators |
|  |  | Electrical production and manipulation equipment |
|  |  | Electronic equipment |
|  |  | Electronic media |
|  |  | Engines |
|  |  | Fertilisers |
|  |  | Food and beverage industry machinery |
|  |  | General construction supplies |
|  |  | General purpose machinery |
|  |  | Glass |
|  |  | Horticultural equipment |
|  |  | Industrial coatings and finishings |
|  |  | Tools including machine shops |
|  |  | Lifting and handling equipment |
|  |  | Lubricants and lubricating equipment |
|  |  | Marine equipment including boats and ships |
|  |  | Measurement and inspection equipment |
|  |  | Medical equipment, supplies and pharmaceuticals |
|  |  | Metals manufacturers, fabricators and stockholders |
|  |  | Moulds, dies and castings |
|  |  | Office and shop equipment |
|  |  | Ovens and furnaces |
|  |  | Packaging |
|  |  | Paints, varnishes and lacquers |
|  |  | Pesticides |
|  |  | Printing related machinery |
|  |  | Published goods |
|  |  | Pumps and compressors |
|  |  | Radar and telecommunications equipment |
|  |  | Road maintenance equipment |
|  |  | Ropes, nets and cordage |
|  |  | Rubber, silicones and plastics |
|  |  | Seals, tapes, taps and valves |
|  |  | Signs |
|  |  | Special purpose machinery and equipment |
|  |  | Textiles, fabrics, silk and machinery |
|  |  | Stationery, stamps, tags and labels |
|  |  | General manufacturing |
|  |  | Vehicle bodybuilders |
|  |  | Vehicle components |
|  |  | Vehicles |
|  |  | Wood products including charcoal, paper, card and board |
|  |  | Workwear |
|  |  | Educational equipment and supplies |
|  |  | Ice |
|  |  | Fences, gates and railings |
|  |  | Access equipment |
|  |  | Car ports and steel buildings |
|  |  | Waste collection, processing and disposal equipment |
|  |  | Glass fibre services |
|  |  | Shelving, storage, safes and vaults |
| Retail | |  |
|  | Clothing and accessories | Clothing |
|  |  | Footwear |
|  |  | Jewellery and fashion accessories |
|  |  | Lingerie and hosiery |
|  |  | Baby and nursery equipment and children's clothes |
|  | Food, drink and multi item retail | Bakeries |
|  |  | Butchers |
|  |  | Confectioners |
|  |  | Delicatessens |
|  |  | Fishmongers |
|  |  | Frozen foods |
|  |  | Green and new age goods |
|  |  | Grocers, farm shops and pick your own |
|  |  | Herbs and spices |
|  |  | Alcoholic drinks including off licences and wholesalers |
|  |  | Organic, health, gourmet and kosher foods |
|  |  | Convenience stores and independent supermarkets |
|  |  | Livestock markets |
|  |  | Markets |
|  |  | Cash and carry |
|  |  | Tea and coffee merchants |
|  |  | Supermarket chains |
|  | Household, office, leisure and garden | Books and maps |
|  |  | Carpets, rugs, soft furnishings and needlecraft |
|  |  | China and glassware |
|  |  | Cosmetics, toiletries, perfumes and hairdressing supplies |
|  |  | Craft supplies |
|  |  | Cycles and accessories |
|  |  | DIY and home improvement |
|  |  | Furniture |
|  |  | Garden centres and nurseries |
|  |  | Garden machinery and furniture |
|  |  | General household goods |
|  |  | Hobby, sports and pastime products |
|  |  | Leather goods, luggage and travel accessories including handbags |
|  |  | Lighting |
|  |  | Music and video |
|  |  | Musical instruments |
|  |  | Pets, supplies and services |
|  |  | Camping and caravanning |
|  |  | Travel agencies |
|  |  | Department stores |
|  |  | Discount stores |
|  |  | Mail order and catalogue stores |
|  |  | Shopping centres and retail parks |
|  |  | Surplus goods |
|  |  | Art and antiques |
|  |  | Charity shops |
|  |  | Florists |
|  |  | Gifts and cards |
|  |  | Party goods and novelties |
|  |  | Secondhand goods |
|  |  | Computer supplies |
|  |  | Domestic appliances |
|  |  | Electrical goods and components |
|  |  | Photographic and optical equipment |
|  |  | Stationery and office supplies |
|  |  | Telephones and telephone cards |
|  |  | Post offices |
|  |  | Garages, garden and portable buildings |
|  |  | Fuel distributors and suppliers |
|  |  | Adult shops |
|  |  | Comics bookshops |
|  |  | Computer shops |
|  |  | Potteries |
|  | Motoring | New vehicles |
|  |  | Secondhand vehicles |
|  |  | Vehicle auctions |
|  |  | Vehicle parts and accessories |
| Transport | |  |
|  | Air | Aeronautical features |
|  |  | Airports and landing strips |
|  |  | Helipads |
|  | Road and rail | Bridges |
|  |  | Cattle grids |
|  |  | Fords and level crossings |
|  |  | Motorway service stations |
|  |  | Parking |
|  |  | Petrol and fuel stations |
|  |  | Roadside telephone boxes |
|  |  | Signalling facilities |
|  |  | Tunnels |
|  |  | Viaducts |
|  |  | Weighbridges |
|  | Walking | Finger posts, guide posts and cairns |
|  |  | Footbridges |
|  |  | Stepping stones |
|  |  | Subways |
|  | Water | Aqueducts |
|  |  | Locks |
|  |  | Moorings and unloading facilities |
|  |  | Rivers and canal organisations and infrastructure |
|  |  | Weirs, sluices and dams |
|  |  | Ferries and ferry terminals |
|  | Public transport, stations and infrastructure | Bus and coach stations, depots and companies |
|  |  | Railway stations, junctions and halts |
|  |  | Tram, metro and light railway stations and stops |
|  |  | Taxi ranks |
|  |  | Underground network stations |
|  |  | London underground entrances |
|  | Bus transport | Bus stops |
|  |  | Hail and ride zones |

*Note*. Group names are underlined; sub-group names are not underlined, and are indented. Adapted from Ordnance Survey (2013).

# Additional Methodological Details

## Re-ranking of LSOAs for relative deprivation

As the degree of deprivation in England is not evenly distributed across urban and rural areas (e.g. only 0.8% of rural LSOAs, versus 12.0% of urban LSOAs are within the lowest decile of deprivation), Index of Multiple Deprivation (IMD) rankings were modified to reflect the degree of deprivation of an LSOA relative to other LSOAs with the same rural/urban classification. This was achieved by first stratifying all LSOAs by their Rural/Urban Classification (RUC) codes into urban and rural environments (RUC codes A1, B1, C1, C2 and D1, D2, E1, E2 respectively). The urban and rural LSOAs were then re-ranked separately, based on their England-wide IMD rankings. Figure 1 illustrates the re-ranking process for six fictional LSOAs. LSOAs were divided into deciles of deprivation based on their new urban/rural IMD ranks.


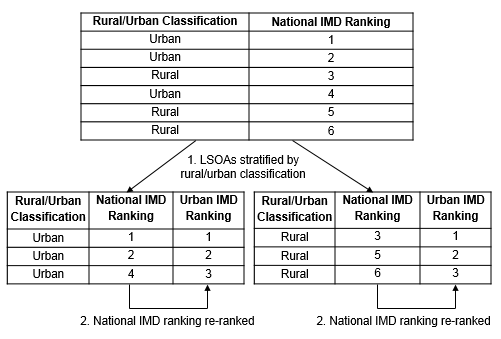


*Figure 1.* Procedure for re-ranking national IMD rankings to urban and rural IMD rankings. Each row represents one LSOA. Step 1: LSOAs are stratified based on their rural/urban classification. Step 2: For urban and rural LSOAs separately, urban/rural rankings are assigned based on national IMD ranks.

## LSOAs Selected for Auditing

Table 3

*Lower Super Output Areas Audited by Area Type, IMD Decile and RUC Classification*

| **Area Type** | **LSOA Code** | **Urban/Rural IMD Decile** | **National IMD decile** | **RUC Class** |
| --- | --- | --- | --- | --- |
| Urban Deprived | Leeds 063D | 2 | 2 | A1 |
|  | Leeds 056E | 2 | 1 | A1 |
|  | Leeds 056C | 2 | 2 | A1 |
|  | Leeds 071C | 1 | 1 | A1 |
|  | Leeds 071B | 1 | 1 | A1 |
|  | Leeds 048A | 1 | 1 | A1 |
|  | Leeds 048D | 1 | 1 | A1 |
|  | Leeds 048C | 1 | 1 | A1 |
|  | Leeds 053B | 1 | 1 | A1 |
|  | Leeds 053C | 1 | 1 | A1 |
|  | Leeds 065A | 1 | 1 | A1 |
| Urban Middle | Leeds 009A | 7 | 7 | A1 |
|  | Leeds 034C | 5 | 5 | A1 |
|  | Leeds 111A | 5 | 5 | A1 |
|  | Leeds 111E | 6 | 5 | A1 |
| Urban Affluent | Leeds 021C | 8 | 8 | A1 |
|  | Leeds 027B | 10 | 10 | A1 |
|  | Leeds 028E | 9 | 9 | A1 |
|  | Leeds 014B | 9 | 9 | A1 |
|  | Leeds 014D | 9 | 9 | A1 |
|  | Leeds 020C | 8 | 8 | A1 |
|  | Leeds 008A | 10 | 10 | A1 |
| Rural Deprived | County Durham 046A | 1 | 3 | D1 |
|  | County Durham 051D | 1 | 1 | D1 |
|  | County Durham 066A | 2 | 4 | D1 |
|  | County Durham 059C | 1 | 2 | D1 |
|  | County Durham 059D | 1 | 2 | D1 |
|  | County Durham 038B | 1 | 2 | D1 |
|  | County Durham 038E | 1 | 2 | D1 |
|  | North Kesteven 007D | 2 | 5 | D1 |
| Rural Middle | County Durham 066C | 6 | 7 | D1 |
|  | County Durham 033A | 6 | 7 | D1 |
|  | Calderdale 004E | 4 | 6 | D1 |
|  | Calderdale 007A | 5 | 7 | D1 |
|  | North Kesteven 004C | 6 | 7 | D1 |
|  | Calderdale 027C | 5 | 6 | D1 |
| Rural Affluent | Leeds 005B | 10 | 10 | D1 |
|  | Leeds 005D | 10 | 10 | D1 |
|  | Leeds 030A | 9 | 9 | D1 |
|  | Leeds 022C | 8 | 8 | D1 |
|  | Leeds 007A | 10 | 10 | D1 |
|  | Leeds 007C | 9 | 9 | D1 |
|  | Leeds 007F | 10 | 10 | D1 |
|  | North Kesteven 006B | 9 | 9 | D1 |
|  | North Kesteven 006D | 9 | 9 | E1 |
|  | North Kesteven 009C | 8 | 9 | D1 |
|  | North Kesteven 001A | 9 | 9 | D1 |
|  | North Kesteven 001B | 10 | 10 | D1 |
|  | North Kesteven 001C | 10 | 10 | D1 |
|  | North Kesteven 001D | 9 | 9 | D1 |
|  | North Kesteven 001E | 10 | 10 | D1 |
|  | Calderdale 027A | 10 | 10 | D1 |
| *Note*. A1: urban major conurbation; D1: rural town and fringe; E1: Rural village and dispersed | | | | |

## Modifications to LSOA boundaries

Once selected, the LSOA boundaries were copied by hand onto printed street maps (Ordnance Survey, 2008; Ordnance Survey, 2015c; Ordnance Survey, 2016a) to define audit areas. The LSOA boundaries were simplified such that each LSOA only included whole road segments (defined as a segment of road running between junctions or notable geographic features such as the edge of a park). This was so that the auditors would easily be able to determine the extent of an audit area by identifying the junction/geographic feature marking the end of the street segment. In general, a road segment that fell partially within the LSOA was included if more than 50% of the segment fell within the LSOA (assessed visually) and was excluded otherwise. However, some roads had to be excluded for safety reasons e.g. if the road was fast and narrow with no footpath and thus could not be walked safely. Furthermore, occasionally additional streets falling outside the LSOA were included within an audit area if (i) one or more food outlets were indicated to be located on the street in close proximity to the LSOA boundary, (ii) to improve efficiency of the audits e.g. if the audit team would need to cover the street anyway, or would need to back-track if the street was not included. This was done to ensure auditing was as efficient as possible, and maximised the number of food outlets identified relative to the financial and time cost involved.

Figure 2 shows an example of two LSOA boundaries and corresponding audit areas to illustrate how LSOA boundaries were modified (note the street maps used during the audits had a higher level of detail than the street maps shown). While the audit areas were not strictly confined to LSOA boundaries, no audit area boundary deviated so substantially from the LSOA boundaries that the environment type classification (e.g. ‘urban deprived’) was likely to be invalid.


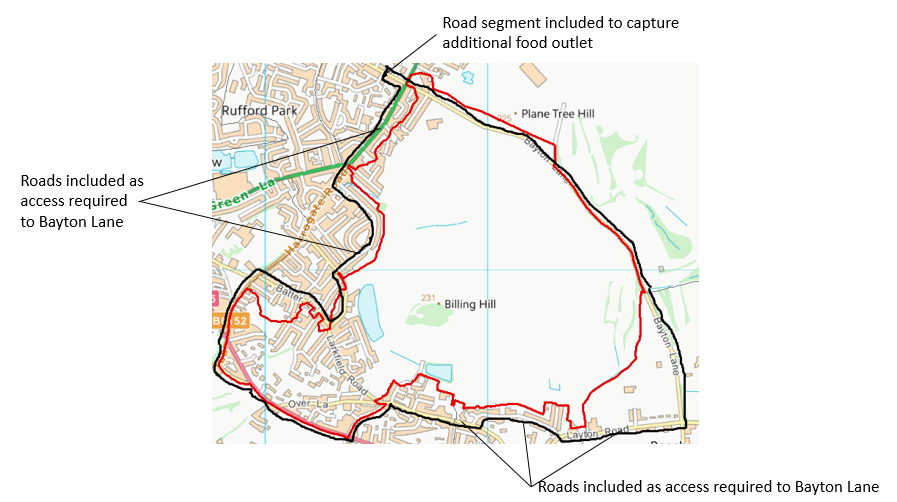


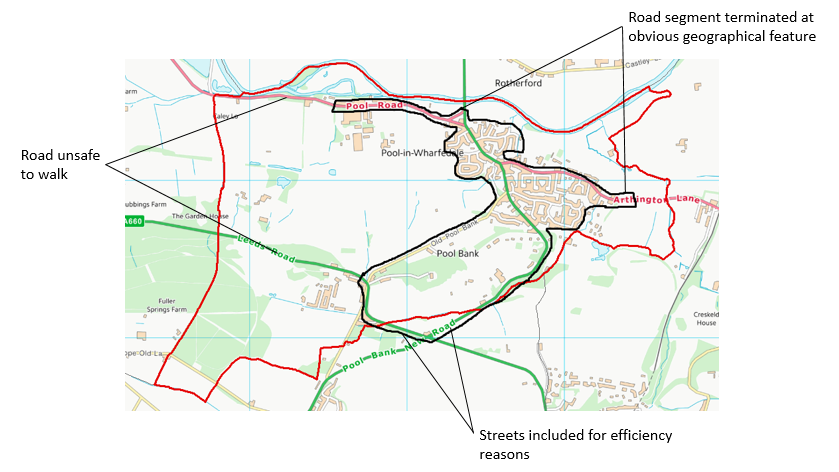


*Figure 2*. Maps showing LSOA boundaries (red) and audit area boundaries (black). Reasons for inclusion/exclusion of street segments also shown. LSOA boundary data from Office for National Statistics (2011). Base-map from Ordnance Survey (2016a).

## Cleaning duplicates

Data cleaning was performed after the data matching process. Entries coded as FP (i.e. false positives - entries within the secondary data, but not identified in the audits) were visually examined to identify duplicates. Duplicates were identified as any two (or more) outlets within an Expected Outlets List (either POI or FSA) with substantially similar outlet names and matching addresses, with agreement between the geographical coordinates of the outlets also being checked when names deviated (e.g. ‘Coriander Cuisine’ and ‘Curriander Cuisine’). If a pair of duplicate entries in the Expected Outlets List was determined to match an outlet in the Audit List, then the duplicate entry whose proprietary classification best matched the broad classification of the Audit List entry was coded as a true positive and the other was coded as a false positive. For example, for a pair of duplicate entries respectively classified as ‘Cafés, snack bars and tea rooms’ and ‘Delicatessen’ in the POI data, and matched to an outlet classified as ‘Café’ in the audits, the first entry would be coded as a true positive, and the second was identified as a duplicate and deleted.

## Audit Classification Scheme

| **1. Restaurant** | | | | | | | | | | |
| --- | --- | --- | --- | --- | --- | --- | --- | --- | --- | --- |
| **1.01** | | | Traditional | | | | | Sit down restaurant | | |
|  | | |  | | | | | Waiter/waitress takes your order | | |
|  | | |  | | | | | Pay for meal after eating | | |
| **1.02** | | | Buffet | | | | | Sit down restaurant | | |
|  | | |  | | | | | No waiter service | | |
|  | | |  | | | | | May pay at the till after food has been selected from the buffet but before eating | | |
|  | | |  | | | | | If 'all you can eat' at a fixed price may pay before or after consumption. Drinks may or may not be included in the price. | | |
| **1.03** | | | Restaurant with takeaway/delivery option | | | | | Primarily a restaurant but has the option to order for takeout | | |
|  | | |  | | | | | Waitress/ waiter service or Food is ordered and paid for at the counter and eaten elsewhere | | |
|  | | |  | | | | | Usually open after 5pm | | |
|  | | |  | | | | | Examples include Chinese restaurants, Indian restaurants, pizza hut | | |
| **1.04** | | | Fast Casual (e.g. Nandos) | | | | | Order and pay for food at counter | | |
|  | | |  | | | | | Waitress/ waiter delivers food to table | | |
|  | | |  | | | | | Similar to fast food but offers a higher quality of food and atmosphere | | |
|  | | |  | | | | | Usually sit down but may have takeaway option | | |
| **1.05** | | | Pub Sit down restaurant | | | | | Sells predominantly alcohol | | |
|  | | |  | | | | | Sit down restaurant | | |
|  | | |  | | | | | Waiter/waitress takes your order | | |
|  | | |  | | | | | Pay for meal after eating | | |
| **1.06** | | | Pub Fast casual | | | | | Sells predominantly alcohol | | |
|  | | |  | | | | | Order and pay for food at bar. Waitress/ waiter delivers food to table | | |
|  | | |  | | | | | Similar to fast food but offers a higher quality of food and atmosphere | | |
|  | | |  | | | | | Sit down only not takeaway | | |
| **1.07** | | | Pub with takeaway/delivery option | | | | | Primarily a pub but has the option to order for takeout | | |
|  | | |  | | | | | Waitress/ waiter service or food is ordered and paid for at the counter and eaten elsewhere | | |
| **1.08** | | | Traditional Hotel | | | | | Restaurant with waiter service | | |
|  | | |  | | | | | Light bar meals with/without waiter service | | |
|  | | |  | | | | | Room service and banqueting rooms | | |
|  | | |  | | | | | May have a buffet for selected meals (e.g. breakfast) | | |
| **2. Pub** | | | | | | | | | | |
| **2.01** | | | Pub no food | | | | | Only alcoholic and non- alcoholic drinks served. | | |
|  | | |  | | | | | May served crisps and nuts behind the bar | | |
|  | | |  | | | | | Includes nightclubs | | |
| **3. Cafe** | | | | | | | | |  |  |
| **3.01** | Traditional café | | | | | Predominantly coffee and hot beverages sold | | |  |  |
|  |  | | | | | Informal seating area | | |  |  |
|  |  | | | | | May have waiter service or order at the counter | | |  |  |
|  |  | | | | | Pre-made/made to order sandwiches and confectionery available | | |  |  |
| **3.02** | Greasy spoon types cafe | | | | | Predominately less healthy fried foods | | |  |  |
|  |  | | | | | Informal seating area | | |  |  |
|  |  | | | | | May have waiter service or order at the counter | | |  |  |
| **3.03** | Specialist café | | | | | Includes milkshake/smoothie bars and ice cream shops | | |  |  |
|  |  | | | | | Similar in style to cafes and coffee shops | | |  |  |
|  |  | | | | | Informal seating area | | |  |  |
|  |  | | | | | Fair trade cafes/coffee shops are included here | | |  |  |
| **3.04** | Café with delicatessen/bakery | | | | | Predominantly café with delicatessen/bakery counter enabling ready-to-eat foods to be taken away | | |  |  |
|  |  | | | | | Informal seating area | | |  |  |
| **3.05** | Sit-in sandwich shop | | | | | Small seating area | | |  |  |
|  |  | | | | | Order and pay at the counter | | |  |  |
|  |  | | | | | Made to order sandwiches/salads etc. May sell drinks, branded snacks and homemade cakes | | |  |  |
|  |  | | | | | No waiter service | | |  |  |
|  |  | | | | | Sit down or takeaway | | |  |  |
| **4. Fast Food** | | | | | | | | |  |  |
| **4.01** | Takeaway café | | | | | Predominantly coffee and hot beverages sold | | |  |  |
|  |  | | | | | No seating - takeaway only | | |  |  |
|  |  | | | | | Pre-made/made to order sandwiches and confectionery available | | |  |  |
| **4.02** | Greasy spoon types cafe | | | | | Predominately less healthy fried foods | | |  |  |
|  |  | | | | | No seating - takeaway only | | |  |  |
| **4.03** | Specialist café | | | | | Includes milkshake/smoothie bars and ice cream shops | | |  |  |
|  |  | | | | | Similar in style to cafes and coffee shops | | |  |  |
|  |  | | | | | Takeaway only | | |  |  |
|  |  | | | | | Fair trade cafes/coffee shops are included here | | |  |  |
| **4.04** | Traditional sandwich shop | | | | | Made to order sandwiches/salads etc. | | |  |  |
|  |  | | | | | May sell drinks, branded snacks and homemade cakes | | |  |  |
|  |  | | | | | No sit in option - takeaway only | | |  |  |
| **4.05** | Internet Cafe | | | | |  | | |  |  |
| **5.01** | Baker - Retail | | | | | Freshly baked savouries/bread, pre-made sandwiches, baked sweet products and branded products | | |  |  |
|  |  | | | | | Usually a chain e.g. Greggs, Milligan's, Bakers Oven but can be independent | | |  |  |
| **6.01** | Traditional takeaway | | | | | | Hot food ordered and paid for at the till | |  |  |
|  |  | | | | | | Wait whilst food is prepared and cooked | |  |  |
|  |  | | | | | | No sit down option to eat-in but may have a seated waiting area. | |  |  |
|  |  | | | | | | Usually open after 5pm | |  |  |
| **6.02** | Traditional takeaway + delivery/collection | | | | | | As traditional plus: The option to telephone for delivery and/or collection | |  |  |
| **6.03** | Traditional takeaway + delivery/collection | | | | | | As traditional plus: Limited seating is available giving the option to eat-in | |  |  |
|  | With seating | | | | | | May have the option to telephone for delivery and/or collection | |  |  |
| **6.04** | Instant fast food | | | | | | Food ordered and paid for at the till | |  |  |
|  |  | | | | | | Available instantly as commonly cooked in bulk in advance and kept hot. Food that can be eaten without cutlery | |  |  |
|  |  | | | | | | Sit down, takeaway and drive-thru facilities | |  |  |
|  |  | | | | | | May be part of a chain or franchise | |  |  |
| **7. Supermarket** | | | | | | | | |  |  |
| **7.01** | Large multiple | | | Large, departmentalised, self-service food store selling food and household goods | | | | |  |  |
|  |  | | | E.g. Tesco, Asda, Morrisons, Sainsburys, Co-op (large), M&S Simply Food (large), Waitrose | | | | |  |  |
| **7.02** | Discount | | | E.g. Kwiksave, Netto, Lidl, Aldi, Farmfoods, Fultons Foods, Iceland | | | | |  |  |
| **7.03** | Small multiple | | | Smaller, self-service food store selling a limited range of food and household goods for greater convenience | | | | |  |  |
|  |  | | | Provides a wider and more consistent supply of fresh produce (e.g. fruits, vegetables, meats, dairy) than traditional convenience stores. | | | | |  |  |
|  |  | | | Not restricted by Sunday trading hours laws. | | | | |  |  |
|  |  | | | Includes small ‘local’ retailers owned by large multiple companies: Tesco metro/express, Sainsbury’s Local, Little Waitrose, Morrison’s My Local, Budgens, Co-op (small), M&S Simply Food (small)  Also includes large chain convenience retailers e.g. Nisa/Premier/Spar/Best-One/Costcutter/Londis. | | | | |  |  |
| **8. Convenience** | | | | | | | | |  |  |
| **8.01** | Traditional (corner shop) | | | Sells groceries, newspapers/magazines, snacks, drinks, lottery, tobacco products and sometimes pre-packed sandwiches | | | | |  |  |
|  |  | | | Small and usually independently owned, although includes small Nisa/Premier/Spar | | | | |  |  |
|  |  | | | Usually have extended hours | | | | |  |  |
|  |  | | | Usually found in more residential areas | | | | |  |  |
| **8.02** | Newsagents | | | Small in size | | | | |  |  |
|  |  | | | Sells primarily newspapers, magazines, snacks, drinks and tobacco products | | | | |  |  |
|  |  | | | In well-trafficked public places | | | | |  |  |
| **8.03** | Petrol Station Shop | | | Sells groceries, newspapers/magazines, snacks, drinks, lottery, tobacco products and sometimes pre-packed sandwiches | | | | |  |  |
|  |  | | | Usually have extended hours | | | | |  |  |
|  |  | | | May be a small multiple supermarket | | | | |  |  |
| **8.04** | Off-licence | | | Licensed to sell alcoholic beverages for consumption off the premises | | | | |  |  |
|  |  | | | Also sells groceries, newspapers, magazines, snacks, drinks and tobacco products. | | | | |  |  |
| **9. Speciality** (Purchase to takeaway only, includes permanent market stalls – e.g. a market stall selling fruits/vegetables should be classed as a greengrocer) | | | | | | | | | |  |
| **9.01** | | Organic food stores | | |  | | | | |  |
| **9.02** | | Health food stores | | | Health supplements | | | | |  |
|  | |  | | | No fresh foods | | | | |  |
| **9.03** | | Fair Trade stores | | |  | | | | |  |
| **9.05** | | Artisan Food Stores | | | Stores selling only locally produced goods | | | | |  |
| **9.06** | | Delicatessen | | | Grocery type store. | | | | |  |
|  | |  | | | Sells fresh ready-to-eat foods (made to order sandwiches/salads, cooked meats and cheeses etc.) | | | | |  |
| **9.07** | | Wine Merchant | | | E.g. Majestic, Oddbins | | | | |  |
| **9.08** | | World food (All sizes) | | | E.g. Oriental, Indian and Continental shops and supermarkets | | | | |  |
| **9.09** | | Candy/sweet/ chocolate shops | | | Shops that do not fall under the category of convenience or confectioners as sell only bought in sweets | | | | |  |
| **9.10** | | Butcher | | | Fresh meat is prepared and sold in store | | | | |  |
| **9.11** | | Baker | | | Bread and baked products prepared fresh and sold in store | | | | |  |
|  | |  | | | Usually independent bakeries | | | | |  |
| **9.12** | | Fishmonger | | | Fresh fish is prepared and sold in store | | | | |  |
| **9.13** | | Greengrocer | | | Sells fresh fruit and vegetables | | | | |  |
| **9.14** | | Dry goods only/Weigh house | | | Dry good only, usually sold by weight | | | | |  |

## Example of allowable street naming discrepancy

Figure 3 shows an example of when a street naming discrepancy would be allowed when matching outlets found in the audits to outlets listed in the secondary data. In this example, an outlet listed in the secondary data as being located on Armley Road would be matched to an outlet having the same name/classification that was found in the audits to be located on Canal Street.


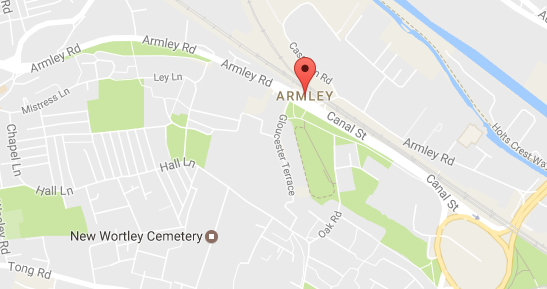


*Figure 3*. Map showing example of street having multiple names (‘Armley Road’ and ‘Canal Street’) [55].

# Additional Descriptive Statistics

Table 4

*Counts of outlets within the audits and secondary datasets and corresponding sensitivity and PPVs for each LSOA based on relaxed matching criteria.*

| LSOA Name | Audits | POI | | | | FSA | | | |
| --- | --- | --- | --- | --- | --- | --- | --- | --- | --- |
|  | Count | | Count | Sens | PPV | | FSA | Sens | PPV |
| **County Durham** | 199 | | 187 | 0.81 | 0.86 | | 197 | 0.90 | 0.91 |
| C. Dur - 033A | 22 | | 18 | 0.82 | 1.00 | | 22 | 0.91 | 0.91 |
| C. Dur - 038B | 5 | | 4 | 0.80 | 1.00 | | 5 | 1.00 | 1.00 |
| C. Dur - 038E | 5 | | 5 | 1.00 | 1.00 | | 6 | 1.00 | 0.83 |
| C. Dur - 046A | 48 | | 45 | 0.79 | 0.84 | | 49 | 0.92 | 0.90 |
| C. Dur - 051D | 26 | | 31 | 0.85 | 0.71 | | 27 | 0.96 | 0.93 |
| C. Dur - 059C | 20 | | 16 | 0.80 | 1.00 | | 18 | 0.90 | 1.00 |
| C. Dur - 059D | 18 | | 15 | 0.67 | 0.80 | | 16 | 0.83 | 0.94 |
| C. Dur - 066A | 47 | | 43 | 0.83 | 0.91 | | 46 | 0.87 | 0.89 |
| C. Dur - 066C | 8 | | 10 | 0.88 | 0.70 | | 8 | 0.88 | 0.88 |
| **Calderdale** | 105 | | 86 | 0.77 | 0.94 | | 93 | 0.81 | 0.90 |
| Calderdale 004E | 63 | | 56 | 0.84 | 0.95 | | 55 | 0.81 | 0.91 |
| Calderdale 007A | 19 | | 14 | 0.63 | 0.86 | | 16 | 0.74 | 0.88 |
| Calderdale 027A | 7 | | 5 | 0.71 | 1.00 | | 8 | 1.00 | 0.88 |
| Calderdale 027C | 16 | | 11 | 0.69 | 1.00 | | 14 | 0.81 | 0.93 |
| **Leeds** | 795 | | 768 | 0.81 | 0.84 | | 726 | 0.83 | 0.91 |
| Leeds - 005B | 22 | | 14 | 0.64 | 1.00 | | 19 | 0.86 | 1.00 |
| Leeds - 005D | 2 | | 2 | 1.00 | 1.00 | | 1 | 0.50 | 1.00 |
| Leeds - 007A | 5 | | 3 | 0.60 | 1.00 | | 3 | 0.60 | 1.00 |
| Leeds - 007C | 10 | | 9 | 0.90 | 1.00 | | 10 | 1.00 | 1.00 |
| Leeds - 007F | 3 | | 2 | 0.67 | 1.00 | | 2 | 0.67 | 1.00 |
| Leeds - 008A | 13 | | 12 | 0.92 | 1.00 | | 14 | 0.85 | 0.79 |
| Leeds - 009A | 36 | | 30 | 0.69 | 0.83 | | 30 | 0.81 | 0.97 |
| Leeds - 014B | 15 | | 12 | 0.73 | 0.92 | | 16 | 1.00 | 0.94 |
| Leeds - 014D | 11 | | 11 | 0.64 | 0.64 | | 8 | 0.64 | 0.88 |
| Leeds - 020C | 26 | | 28 | 0.92 | 0.86 | | 20 | 0.73 | 0.95 |
| Leeds - 021C | 44 | | 40 | 0.86 | 0.95 | | 38 | 0.82 | 0.95 |
| Leeds - 022C | 3 | | 4 | 1.00 | 0.75 | | 3 | 0.67 | 0.67 |
| Leeds - 027B | 25 | | 23 | 0.84 | 0.91 | | 23 | 0.88 | 0.91 |
| Leeds - 028E | 17 | | 15 | 0.88 | 1.00 | | 17 | 0.94 | 0.94 |
| Leeds - 030A | 8 | | 5 | 0.63 | 1.00 | | 8 | 0.88 | 0.88 |
| Leeds - 034C | 42 | | 46 | 0.88 | 0.80 | | 45 | 0.95 | 0.89 |
| Leeds - 048A | 32 | | 40 | 0.88 | 0.70 | | 31 | 0.88 | 0.90 |
| Leeds - 048C | 7 | | 8 | 0.86 | 0.75 | | 8 | 0.86 | 0.75 |
| Leeds - 048D | 44 | | 44 | 0.84 | 0.84 | | 41 | 0.80 | 0.85 |
| Leeds - 053B | 22 | | 23 | 0.77 | 0.74 | | 20 | 0.82 | 0.90 |
| Leeds - 053C | 24 | | 24 | 0.83 | 0.83 | | 19 | 0.79 | 1.00 |
| Leeds - 056C | 7 | | 7 | 1.00 | 1.00 | | 8 | 0.86 | 0.75 |
| Leeds - 056E | 3 | | 5 | 1.00 | 0.60 | | 4 | 1.00 | 0.75 |
| Leeds - 063D | 31 | | 26 | 0.77 | 0.92 | | 31 | 0.97 | 0.97 |
| Leeds - 065A | 13 | | 11 | 0.85 | 1.00 | | 11 | 0.77 | 0.91 |
| Leeds - 071B | 26 | | 18 | 0.65 | 0.94 | | 22 | 0.85 | 1.00 |
| Leeds - 071C | 40 | | 38 | 0.80 | 0.84 | | 30 | 0.68 | 0.90 |
| Leeds - 111A | 78 | | 85 | 0.86 | 0.79 | | 78 | 0.85 | 0.85 |
| Leeds - 111E | 186 | | 183 | 0.80 | 0.81 | | 166 | 0.82 | 0.92 |
| **North Kesteven** | 73 | | 59 | 0.78 | 0.97 | | 65 | 0.85 | 0.95 |
| North Kesteven 001A | 3 | | 3 | 1.00 | 1.00 | | 3 | 1.00 | 1.00 |
| North Kesteven 001B | 1 | | 1 | 1.00 | 1.00 | | 1 | 1.00 | 1.00 |
| North Kesteven 001C | 8 | | 7 | 0.75 | 0.86 | | 8 | 1.00 | 1.00 |
| North Kesteven 001D | 6 | | 6 | 0.83 | 0.83 | | 5 | 0.83 | 1.00 |
| North Kesteven 001E | 5 | | 3 | 0.60 | 1.00 | | 5 | 1.00 | 1.00 |
| North Kesteven 004C | 7 | | 5 | 0.71 | 1.00 | | 6 | 0.86 | 1.00 |
| North Kesteven 006B | 12 | | 10 | 0.83 | 1.00 | | 8 | 0.67 | 1.00 |
| North Kesteven 006D | 11 | | 9 | 0.82 | 1.00 | | 8 | 0.64 | 0.88 |
| North Kesteven 007D | 4 | | 2 | 0.50 | 1.00 | | 5 | 1.00 | 0.80 |
| North Kesteven 009C | 16 | | 13 | 0.81 | 1.00 | | 16 | 0.94 | 0.94 |
| *Note*. Sens: sensitivity | | | | | | | | | |

# Additional Tables of Results

## Strict Matching Criteria

Table 5

*Odds of true positive relative to false positive (PPV odds) for POI data with strict matching criteria applied*

| Environment/ Outlet Type | Model 1 | | | Model 2 | | | Model 3 | | |
| --- | --- | --- | --- | --- | --- | --- | --- | --- | --- |
|  | OR | 95% CI | | OR | 95% CI | | OR | 95% CI | |
| Urban | REF |  |  |  |  |  | REF |  |  |
| Rural | **1.86^2^** | **1.26** | **2.88** |  |  |  | **1.69^1^** | **1.00** | **2.92** |
| Deprived |  |  |  | REF |  |  | REF |  |  |
| Middle |  |  |  | 1.26 | 0.81 | 2.02 | 1.16 | 0.68 | 1.86 |
| Affluent |  |  |  | **1.90^2^** | **1.17** | **3.14** | 1.67 | 0.94 | 2.92 |
| Restaurant |  |  |  |  |  |  | REF |  |  |
| Pub |  |  |  |  |  |  | **0.33^3^** | **0.18** | **0.62** |
| Café |  |  |  |  |  |  | 0.73 | 0.44 | 1.24 |
| Fast Food |  |  |  |  |  |  | 0.69 | 0.44 | 1.09 |
| Supermarket |  |  |  |  |  |  | 0.79 | 0.42 | 1.55 |
| Convenience |  |  |  |  |  |  | **0.38^3^** | **0.21** | **0.67** |
| Speciality |  |  |  |  |  |  | 0.80 | 0.43 | 1.53 |
| Rural*Middle |  |  |  |  |  |  | 1.20 | 0.52 | 2.90 |
| Rural*Affluent |  |  |  |  |  |  | 1.39 | 0.52 | 3.86 |
| *Note*. OR: Odds ratio. CI: Confidence interval. REF: Reference category. All models are multi-level models accounting for nesting of outlets within LSOAs. ^1^p<0.05, ^2^p<0.01, ^3^p<0.001 | | | | | | | | | |

Table 6

*Odds of true positive relative to false negative (sensitivity odds) for POI data with strict matching criteria applied*

| Environment/ Outlet Type | Model 1 | | | Model 2 | | | Model 3 | | |
| --- | --- | --- | --- | --- | --- | --- | --- | --- | --- |
|  | OR | 95% CI | | OR | 95% CI | | OR | 95% CI | |
| Urban | REF |  |  |  |  |  | REF |  |  |
| Rural | 0.93 | 0.71 | 1.23 |  |  |  | 1.34 | 0.85 | 2.13 |
| Deprived |  |  |  | REF |  |  | REF |  |  |
| Middle |  |  |  | 1.17 | 0.86 | 1.57 | 1.43 | 0.95 | 2.15 |
| Affluent |  |  |  | 1.09 | 0.77 | 1.54 | 1.40 | 0.87 | 2.28 |
| Restaurant |  |  |  |  |  |  | REF |  |  |
| Pub |  |  |  |  |  |  | 0.61 | 0.33 | 1.15 |
| Café |  |  |  |  |  |  | **0.43^3^** | **0.28** | **0.64** |
| Fast Food |  |  |  |  |  |  | 0.99 | 0.66 | 1.50 |
| Supermarket |  |  |  |  |  |  | 1.98 | 0.99 | 4.34 |
| Convenience |  |  |  |  |  |  | **0.47^2^** | **0.29** | **0.76** |
| Speciality |  |  |  |  |  |  | 0.72 | 0.43 | 1.21 |
| Rural*Middle |  |  |  |  |  |  | 0.61 | 0.32 | 1.18 |
| Rural*Affluent |  |  |  |  |  |  | 0.58 | 0.28 | 1.19 |
| *Note*. OR: Odds ratio. CI: Confidence interval. REF: Reference category. All models are multi-level models accounting for nesting of outlets within LSOAs. ^1^p<0.05, ^2^p<0.01, ^3^p<0.001 | | | | | | | | | |

Table 7

*Odds of true positive relative to false positive (PPV odds) for FSA data with strict matching criteria applied*

| Environment/ Outlet Type | Model 1 | | | Model 2 | | | Model 3 | | |
| --- | --- | --- | --- | --- | --- | --- | --- | --- | --- |
|  | OR | 95% CI | | OR | 95% CI | | OR | 95% CI | |
| Urban | REF |  |  |  |  |  | REF |  |  |
| Rural | 1.45 | 0.99 | 2.22 |  |  |  | 1.91 | 0.97 | 3.88 |
| Deprived |  |  |  | REF |  |  | REF |  |  |
| Middle |  |  |  | 1.18 | 0.77 | 1.85 | 1.29 | 0.66 | 2.49 |
| Affluent |  |  |  | 1.47 | 0.90 | 2.48 | 1.42 | 0.70 | 2.91 |
| Restaurant |  |  |  |  |  |  | REF |  |  |
| Pub |  |  |  |  |  |  | **0.20^3^** | **0.10** | **0.42** |
| Café |  |  |  |  |  |  | **0.53^1^** | **0.28** | **1.00** |
| Fast Food |  |  |  |  |  |  | 1.00 | 0.52 | 1.91 |
| Supermarket |  |  |  |  |  |  | 2.30 | 0.75 | 10.11 |
| Convenience |  |  |  |  |  |  | **0.19^3^** | **0.10** | **0.35** |
| Speciality |  |  |  |  |  |  | 0.54 | 0.25 | 1.19 |
| Rural*Middle |  |  |  |  |  |  | 0.69 | 0.24 | 1.98 |
| Rural*Affluent |  |  |  |  |  |  | 1.06 | 0.33 | 3.49 |
| *Note*. OR: Odds ratio. CI: Confidence interval. REF: Reference category. All models are multi-level models accounting for nesting of outlets within LSOAs. ^1^p<0.05, ^2^p<0.01, ^3^p<0.001 | | | | | | | | | |

Table 8

*Odds of true positive relative to false negative (sensitivity odds) for FSA data with strict matching criteria applied*

| Environment/ Outlet Type | Model 1 | | | Model 2 | | | Model 3 | | | Model 3 (urban only) | | | Model 3 (rural only) | | |
| --- | --- | --- | --- | --- | --- | --- | --- | --- | --- | --- | --- | --- | --- | --- | --- |
|  | OR | 95% CI | | OR | 95% CI | | OR | 95% CI | | OR | 95% CI | | OR | 95% CI | |
| Urban | REF |  |  |  |  |  | REF |  |  |  |  |  |  |  |  |
| Rural | **1.42^1^** | **1.01** | **1.99** |  |  |  | **2.55^2^** | **1.47** | **4.57** |  |  |  |  |  |  |
| Deprived |  |  |  | REF |  |  | REF |  |  | REF |  |  | REF |  |  |
| Middle |  |  |  | 1.05 | 0.69 | 1.60 | 1.27 | 0.81 | 2.05 | 1.25 | 0.78 | 2.17 | **1.25^1^** | **0.27** | **0.98** |
| Affluent |  |  |  | 0.98 | 0.64 | 1.50 | 1.15 | 0.70 | 1.92 | 1.13 | 0.67 | 1.91 | 1.13 | 0.33 | 1.29 |
| Restaurant |  |  |  |  |  |  | REF |  |  | REF |  |  | REF |  |  |
| Pub |  |  |  |  |  |  | **0.51^1^** | **0.26** | **1.01** | **0.34^2^** | **0.15** | **0.77** | 0.34 | 0.38 | 5.93 |
| Café |  |  |  |  |  |  | **0.61^1^** | **0.38** | **0.98** | 0.58 | 0.33 | 1.03 | 0.58 | 0.31 | 1.58 |
| Fast Food |  |  |  |  |  |  | 1.08 | 0.67 | 1.75 | 1.00 | 0.56 | 1.80 | 1.00 | 0.56 | 2.98 |
| Supermarket |  |  |  |  |  |  | 1.68 | 0.78 | 4.05 | 1.29 | 0.54 | 3.46 | 1.29 | 0.78 | 77.39 |
| Convenience |  |  |  |  |  |  | **0.36^3^** | **0.21** | **0.60** | **0.33^2^** | **0.17** | **0.64** | **0.33^1^** | **0.18** | **0.99** |
| Speciality |  |  |  |  |  |  | **0.38^3^** | **0.22** | **0.66** | **0.33^2^** | **0.17** | **0.65** | 0.33 | 0.22 | 1.30 |
| Rural*Middle |  |  |  |  |  |  | **0.40^1^** | **0.18** | **0.86** |  |  |  |  |  |  |
| Rural*Affluent |  |  |  |  |  |  | 0.52 | 0.22 | 1.20 |  |  |  |  |  |  |
| *Note*. OR: Odds ratio. CI: Confidence interval. REF: Reference category. All models are multi-level models accounting for nesting of outlets within LSOAs. ^1^p<0.05, ^2^p<0.01, ^3^p<0.001 | | | | | | | | | | | | | | | |

## Classification Agreement

Table 13

*Percentage Agreement for Broad Classifications Based on Primary and Alternate Classification Schemes*

|  |  | **Points of Interest** | | | | | | |
| --- | --- | --- | --- | --- | --- | --- | --- | --- |
|  |  | Rest | Pub | Café | FF | Sup | Conv | Spec |
| **Audit** | Rest | **120** | 117 | 3 | 5 | 0 | 0 | 0 |
|  | Pub | 3 | **42** | 0 | 0 | 0 | 0 | 0 |
|  | Café | 5 | 2 | **83** | 22 | 0 | 0 | 9 |
|  | FF | 10 | 1 | 7 | **213** | 0 | 0 | 4 |
|  | Sup | 0 | 0 | 0 | 0 | **43** | 22 | 6 |
|  | Conv | 0 | 0 | 0 | 0 | 1 | **69** | 2 |
|  | Spec | 0 | 0 | 0 | 10 | 0 | 13 | **59** |
|  | **%Agree** | **46%** | **26%** | **63%** | **78%** | **60%** | **65%** | **57%** |

*Note*. Rest: Restaurant; FF: Fast Food; Conv: Convenience; Spec: Speciality; %Agree: percentage agreement for broad classifications. Numbers in bold indicate the counts of outlets for which the POI-derived and the audit-derived classifications agreed. Numbers in red indicate the counts of outlets for which the POI-derived and the audit-derived classifications disagreed.
